# Transcriptomic underpinnings of high and low mirror aggression zebrafish behaviours

**DOI:** 10.1186/s12915-022-01298-z

**Published:** 2022-05-02

**Authors:** Florian Reichmann, Johannes Pilic, Slave Trajanoski, William H. J. Norton

**Affiliations:** 1 Division of Pharmacology, Otto Loewi Research Center, Medical University of Graz, Graz, Austria; 2 Department of Genetics and Genome Biology, College of Life Sciences, University of Leicester, Leicester, UK; 3 Center for Medical Research, Medical University of Graz, Graz, Austria; 4 Department of Genetics, Institute of Biology, ELTE Eötvös Loránd University, Budapest, Hungary

**Keywords:** Mirror aggression, Anxiety, High aggression zebrafish, Low aggression zebrafish, RNAseq, Transcriptomics

## Abstract

**Background:**

Aggression is an adaptive behaviour that animals use to protect offspring, defend themselves and obtain resources. Zebrafish, like many other animals, are not able to recognize themselves in the mirror and typically respond to their own reflection with aggression. However, mirror aggression is not an all-or-nothing phenomenon, with some individuals displaying high levels of aggression against their mirror image, while others show none at all. In the current work, we have investigated the genetic basis of mirror aggression by using a classic forward genetics approach - selective breeding for high and low mirror aggression zebrafish (HAZ and LAZ).

**Results:**

We characterized AB wild-type zebrafish for their response to the mirror image. Both aggressive and non-aggressive fish were inbred over several generations. We found that HAZ were on average more aggressive than the corresponding LAZ across generations and that the most aggressive adult HAZ were less anxious than the least aggressive adult LAZ after prolonged selective breeding. RNAseq analysis of these fish revealed that hundreds of protein-encoding genes with important diverse biological functions such as arsenic metabolism (*as3mt*), cell migration (*arl4ab*), immune system activity (*ptgr1*), actin cytoskeletal remodelling (*wdr1*), corticogenesis (*dgcr2*), protein dephosphorylation (*ublcp1*), sialic acid metabolism (*st6galnac3*) and ketone body metabolism (*aacs*) were differentially expressed between HAZ and LAZ, suggesting a strong genetic contribution to this phenotype. DAVID pathway analysis showed that a number of diverse pathways are enriched in HAZ over LAZ including pathways related to immune function, oxidation-reduction processes and cell signalling. In addition, weighted gene co-expression network analysis (WGCNA) identified 12 modules of highly correlated genes that were significantly associated with aggression duration and/or experimental group.

**Conclusions:**

The current study shows that selective breeding based of the mirror aggression phenotype induces strong, heritable changes in behaviour and gene expression within the brain of zebrafish suggesting a strong genetic basis for this behaviour. Our transcriptomic analysis of fish selectively bred for high and low levels of mirror aggression revealed specific transcriptomic signatures induced by selective breeding and mirror aggression and thus provides a large and novel resource of candidate genes for future study.

**Supplementary Information:**

The online version contains supplementary material available at 10.1186/s12915-022-01298-z.

## Background

Aggression is an adaptive behaviour that animals use to protect offspring, defend themselves and obtain resources [1]. However, heightened aggression can cause injury or death meaning that expression of this behaviour must be tightly controlled. Aggression also plays an important role in establishing and maintaining social hierarchies. Within a population, animals will show varying levels of aggression depending upon their dominance status, and intrinsic factors such as size, fitness, or metabolic status [2, 3].

Zebrafish (*Danio rerio*) are small fresh water teleosts that display a characteristic horizontal stripe pattern. They are frequently used as animal models in biomedical research due to their rapid development, genetic amenability and optical transparency at larval stages [4]. In recent years, zebrafish have also been used for behavioural neuroscience. A large number of robust behaviours have been described in both larval and adult fish [5–7], and at young stages, the small size and relative transparency of the brain permit neural activity to be imaged in freely behaving animals [8].

Since aggressive behaviour is an evolutionarily trait, studies in model species such as zebrafish may allow us to uncover biological mechanisms that are conserved across animal species including humans [9]. Zebrafish aggression can be measured in either dyadic fights [10] or using mirror-image stimulation, a reproducible and non-invasive method that has provided insights into the genetic and neurobiological basis of this behaviour [11–13]. Zebrafish do not recognize themselves in the mirror [14] and fight as if another fish is present. They display aggressive postures that include biting the mirror image, thrashing the tail and extending their fins [11]. Zebrafish aggression has a moderate heritability index of 0.36 [13] suggesting that both genes and the environment can influence this behaviour. Candidate gene approaches have shown that multiple signalling pathways can affect aggression. For example, zebrafish *fibroblast growth factor receptor 1a* (*fgfr1a*) and *endothelin receptor type Aa* (*ednraa*) mutants are more aggressive towards their mirror image compared to corresponding wild-type fish [15, 16], whereas both *nitric oxide synthase 1* (*nos1*) and *histamine receptor H3* (*hrh3*) mutants are less aggressive than conspecifics in a mirror aggression test [17, 18]. In addition, strain differences in mirror aggression [19] and gene expression changes in the brain after mirror exposure [20, 21] have identified some of the genetic signalling networks that are important for this behaviour. In a recent study, we have characterized the brain areas that respond to mirror aggression using the neural activation marker rpS6 [18]. This approach demonstrated that many parts of the social decision-making network (SDMN) are stimulated by mirror fighting, in keeping with aggression-induced neuronal activation in the SDMN in other species [22].

Similar to other behaviours, mirror aggression is not an all-or-nothing phenomenon. Instead, some individuals within a group display high levels of aggression against their mirror image and others none at all, representing a normal distribution of behaviour [11, 15, 19, 23, 24]. This variation is likely driven by the genetic and environmental factors that shape the response of an individual towards its mirror image. However, the precise transcriptomic differences between aggressive mirror fighters and non-aggressive individuals during mirror exposure are not known. In the current work, we have investigated the genetic contribution to mirror aggression by using a classic forward genetics approach—selective breeding for high and low mirror aggression zebrafish (HAZ and LAZ). We hypothesized that mirror aggression phenotype is heritable, that behavioural selection and mirror fighting will induce a unique transcriptional signature in the brain, and that high mirror aggression zebrafish will display morphometric differences compared to low aggression zebrafish.

## Results

### High mirror-induced aggression phenotype is heritable over generations

**Fig. 1:**
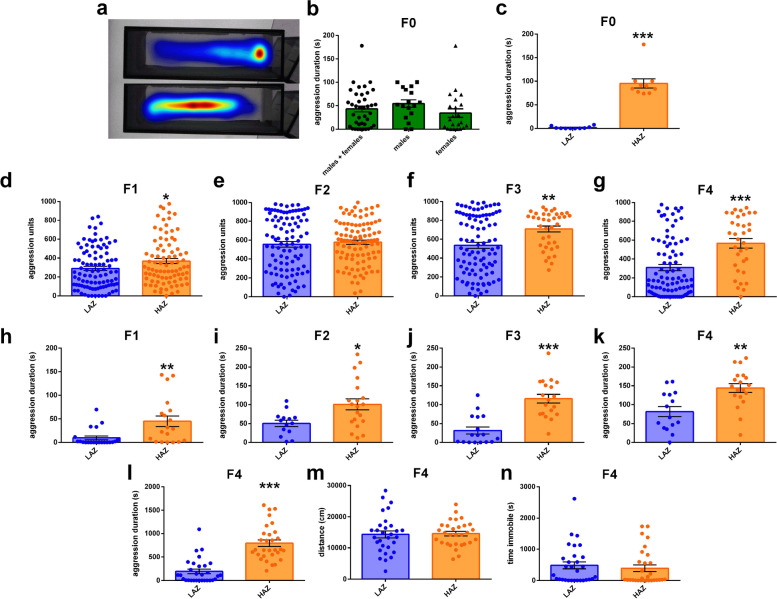
Transgenerational effects of selective breeding on mirror fighting. **a** Representative heatmap images of an aggressive (top part) and non-aggressive (bottom part) zebrafish individual in the mirror-induced aggression (MIA) setup. **b** Variation in time spent interacting with the mirror of arbitrarily-selected male and female zebrafish from the local breeding colony (F0 fish) during a 5 min MIA assay. **c** The 10 most aggressive F0 zebrafish spent significantly more time in aggressive display than the 10 least aggressive F0 zebrafish. *n* = 10/group (**d**–**g**). Increased mirror aggression levels of **d** 1-month-old (*n* = 85–96) F1 HAZ (high aggression zebrafish) derived from F0 HAZ, **e** 1-month-old (*n* = 100/group) F2 HAZ derived from F1 HAZ, **f** 1-month-old (*n* = 38–98) F3 HAZ derived from F2 HAZ and **g** 1-month-old (*n* = 34–90) F4 HAZ derived from F3 HAZ compared to respective LAZ (low aggression zebrafish). **h**–**k** Mirror aggression levels of the most aggressive juvenile HAZ at adulthood (3 months of age) compared to the mirror aggression levels of the least aggressive juvenile LAZ at adulthood. Analysis revealed higher mirror aggression levels of **h** the most aggressive F1 HAZ at 3 months of age (*n* = 19–20), **i** the most aggressive F2 HAZ at 3 months of age (*n* = 14–20), **j** the most aggressive F3 HAZ at 3 months of age (*n* = 17–19) and **k** the most aggressive F4 HAZ at 3 months of age (*n* = 15–19) compared to the least aggressive LAZ of the respective generation at 3 months of age. **l**–**n** Behaviour of adult F4 high aggression zebrafish (HAZ) and low aggression zebrafish (LAZ) during prolonged mirror exposure. **l** Time spent interacting with the mirror, **m** distance travelled and **n** time spent immobile of HAZ and LAZ exposed to a 1h mirror-induced aggression assay (*n* = 30/group). Mann-Whitney *U* test. ***, *P* < 0.001; **, *P* < 0.01 and *, *P* < 0.05 vs. respective LAZ. Data are presented as mean ± SEM. Source data and individual data values are available in Additional file 2

To establish high mirror aggression zebrafish (HAZ) and low mirror aggression zebrafish (LAZ) lines, we randomly selected AB wild-type zebrafish from our local breeding colony and characterized their response to their own mirror image (the F0 generation). These zebrafish exhibited a large variation in aggression ranging from 0 to 178.06 s during a standard 5 min observation period with no differences between males and females (Fig. 1a, b). However, the aggression level of the 10 most aggressive and the 10 least aggressive F0 zebrafish was clearly separable (Fig. 1c). To investigate whether low and/or high mirror-induced aggression is heritable, we measured this behaviour in the offspring of the 3 most aggressive HAZ males and females and the 3 least aggressive LAZ males and females (F1 generation) respectively. Analysis revealed that F1 HAZ are on average more aggressive towards their mirror image than F1 LAZ at 1 month of age (i.e. juvenile zebrafish, Fig. 1d). Although F2 juvenile HAZ and LAZ did not show differences in mirror aggression (Fig. 1e), F3 and F4 juvenile fish did, again with HAZ being on average more aggressive than LAZ (Fig. 1f, g). This suggests that genetic factors are contributing to this behaviour. We also evaluated mirror aggression in young adult fish (3 months of age) across generations. For this, we selected the 20 most aggressive juvenile HAZ in each generation, raised them to adulthood and compared them to the 20 least aggressive juvenile LAZ of the corresponding generation, in order to better characterize the most and least aggressive individuals in each generation and maximize selection for and against mirror aggression over generations. As can be seen in Fig. 1h–k, these preselected adult HAZ were more aggressive than the preselected LAZ in all generations investigated suggesting that the propensity to be aggressive in the mirror test remains stable from adolescence to adulthood. Interestingly, when comparing data across generations, it appears that HAZ (especially preselected adult fish) became more aggressive over generations as suggested by increasing average times spent in an aggressive display. In contrast, LAZ remained stable (juvenile fish) or tended to increase in average aggression levels (adult fish) suggesting that high mirror-induced aggression phenotype, but not low mirror-induced aggression phenotype is heritable over generations. On the other hand, it is also clear that there is a considerable overlap in time spent in mirror aggression between LAZ and HAZ after prolonged selective breeding (F4 generation) with high and low mirror aggression individuals found in both groups (Fig. 1g, k). However, the fraction of non-aggressive or low aggressive individuals is considerably higher in LAZ and the fraction of highly aggressive individuals is markedly greater in HAZ. To analyse how long-term mirror exposure affects aggressive behaviour of F4 HAZ and LAZ, we next recorded 30 mixed sex and size-matched adult HAZ (13 males, 17 females, size: 2.70 +/− 0.05 cm) and LAZ (14 males, 16 females, size: 2.58 +/− 0.03 cm) during a prolonged MIA session (1h). Similar to our findings in the 5-min mirror-induced aggression (MIA) assay (Fig. 1g, k), HAZ were significantly more aggressive than LAZ without changes to locomotor activity (Fig. 1l–n).

### Selective breeding for mirror aggression co-selects for anxiety

**Fig. 2:**
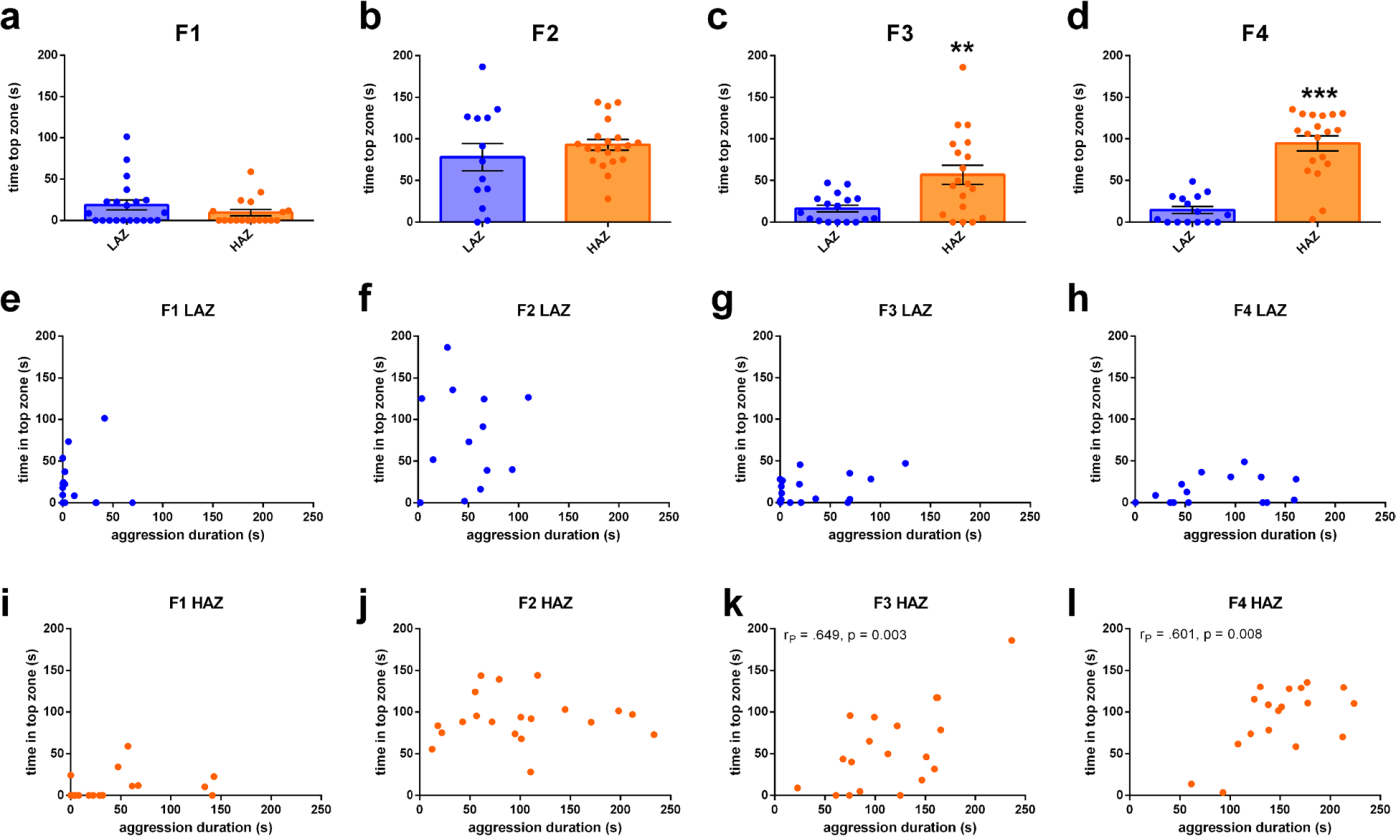
Transgenerational effects of selective breeding on anxiety-like behaviour. **a**–**d** Time spent in the top zone of the novel tank diving test across generations (F1–F4) of adult high aggression zebrafish (HAZ) and adult low aggression zebrafish (LAZ), which had been previously tested in the mirror-induced aggression assay. Mann-Whitney *U* test. *n* = 13–21. ***, *P* < 0.001 and **, *P* < 0.01 vs. LAZ. Data are presented as mean ± SEM. **e**–**h** Scatter plots visualizing Pearson correlation analysis between time spent attacking the mirror and time spent in the top zone of the novel tank diving test of LAZ across generations (F1–F4). *n* = 13–21. **i**–**l** Scatter plots visualizing Pearson correlation analysis between time spent attacking the mirror and time spent in the top zone of the novel tank diving test of HAZ across generations (F1–F4). *n* = 18–20. Significant correlations are shown by displaying the correlation coefficient (*r*_*p*_) and *p* value in the respective Figure panels. Source data and individual data values are available in Additional file 2

To evaluate whether selective breeding for aggression also affects anxiety, the preselected adult HAZ and LAZ that had been tested in the 5-min MIA assay were also tested in the novel tank diving test (NTD [25];) in each generation. Although these fish did not show differences in anxiety-like behaviour in the F1 and F2 generations (Fig. 2a, b), further selective breeding led to changes in anxiety-like behaviour between the groups. Specifically, in the F3 and F4 generations HAZ spent on average more time in the top compartment of the NTD than LAZ indicating reduced anxiety (Fig. 2c, d). To evaluate whether higher aggression levels and lower anxiety levels are correlated, we used Pearson correlation analysis. As expected, there was no correlation between aggression and anxiety in the F1 and F2 generations given that HAZ did not differ from LAZ in anxiety phenotype in these generations (Fig. 2e–j). However, analysis of the F3 and F4 generation revealed a significant negative correlation between aggression duration and time in the top zone for HAZ (Fig. 2k, l). This suggests that aggressive HAZ are also less anxious.

To evaluate whether F4 HAZ and LAZ differ in other behavioural domains as well, these fish were tested for locomotion, social behaviour and boldness. We did not observe any other differences in behaviour including locomotor activity in the open field test (distance moved, velocity, time spent immobile, and angular velocity), social behaviour (shoaling and social interaction) and novel object boldness (Additional file 1: Fig. S1).

### Selective breeding and mirror exposure strongly alter the brain transcriptome

Next, we investigated how selective breeding and mirror fighting affect the brain transcriptome. We collected the brains of HAZ and LAZ founder fish used in this study (F0 generation), brains from F4 HAZ and LAZ individuals not exposed to the mirror and brains from F4 HAZ and LAZ exposed to the mirror for 1h. Analysis of the F0 fish brains collected at baseline revealed only minor transcriptomic differences between HAZ and LAZ founders. Although the two groups were very different during the mirror assay (Fig. 1c), differential expression analysis showed that only 6 protein-coding genes (*prostaglandin reductase 1* (*ptgr1*), *si:ch211-213a13.2*, *secretogranin V* (*scg5*), *si:dkey-238k10.1*, *LON peptidase N-terminal domain and ring finger 1* (*lonrf1*) and *si:ch211-76m11.3*) were differentially expressed (padj < 0.05 and LFC > |2|). In addition, no clustering of the HAZ and LAZ samples was evident by principal component analysis (PCA; Additional file 1: Fig. S2). This suggests that the F0 fish used to generate high aggressive and low aggressive lines were similar in their baseline genetic makeup and that their different behaviour during the MIA assay was most likely the result of non-genetic factors (e.g. tank hierarchy) in the founders.

**Fig. 3:**
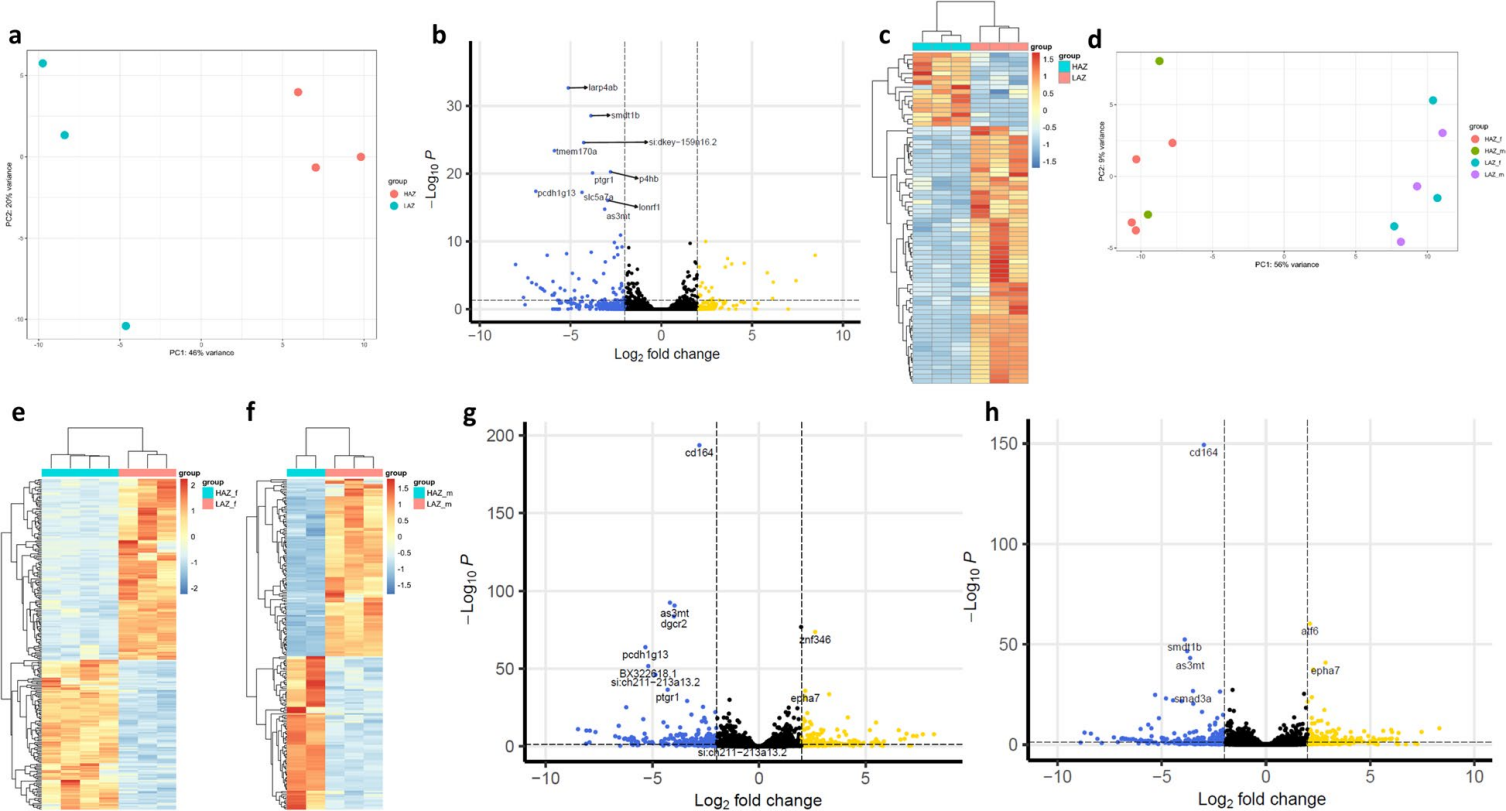
Selective breeding-induced and mirror-induced neurotranscriptomic differences of male and female high aggression zebrafish (HAZ) and low aggression zebrafish (LAZ). **a** Principal component analysis plot of the top 200 most variable genes after differential expression analysis between F4 HAZ and LAZ without mirror exposure. *n* = 3/group. **b** Volcano plot of differentially expressed genes (DEGs; padj < 0.05 and LFC > |2|) between F4 HAZ and LAZ at baseline. DEGs with the lowest adjusted *p* values (padj) are highlighted. *n* = 3/group. **c** Heat map displaying DEGs between F4 HAZ and LAZ at baseline. Hierarchical clustering of samples and genes reveals large differences between HAZ and LAZ, but similar transcriptional patterns within the two lines. *n* = 3/group. **d** Principal component analysis plot of the top 200 most variable genes after differential expression analysis between F4 HAZ and LAZ displaying similar aggression levels after mirror exposure. *n* = 6/group. **e** Heatmap of DEGs between female HAZ (HAZf) and female LAZ (LAZf) after mirror exposure. Hierarchical clustering of samples and genes reveals large differences between HAZ and LAZ, but similar transcriptional patterns within aggression subgroups. *n* = 3-4/group. **f** Heatmap of DEGs between male HAZ (HAZm) and male LAZ (LAZm) after mirror exposure. Hierarchical clustering of samples and genes reveals similar differences like in corresponding male animals. *n*=2–3/group. **g** Volcano plot displaying DEGs between HAZf and LAZf after mirror exposure. DEGs with lowest adjusted p values (padj) are highlighted. *n* = 3–4/group. **h** Volcano plot displaying DEGs between HAZm and LAZm after mirror exposure. DEGs with the lowest adjusted p values (padj) are highlighted. *n* = 2–3/group. Golden dots in Volcano plots indicate genes upregulated in HAZ more than log fold change 2, blue dots represent genes downregulated in HAZ more than LFC − 2 and black dots represent genes not passing these thresholds. Source data and individual data values are available at the ebrains data repository, DOI: 10.25493/VTP5-8J9 and in Additional file 2

When analysing the brain transcriptome of F4 HAZ and LAZ fish, the situation was different. Already at baseline (F4 fish not exposed to a mirror), there were considerable gene expression differences between the groups. PCA revealed a clear separation of HAZ and LAZ along PC1 of the plot explaining 46% of the total variance (Fig. 3a) and differential expression analysis showed 85 differentially expressed genes (DEGs) between the two groups. Twenty-two of these DEGs were upregulated in HAZ compared to LAZ, while 63 DEGs were downregulated (Fig. 3b). Hierarchical clustering of samples also revealed a clear separation between HAZ and LAZ indicating similar transcriptional patterns within the 2 lines (Fig. 3c).

To analyse how baseline gene expression differences between HAZ and LAZ relate to mirror aggression behaviour, we also sequenced F4 LAZ and HAZ brain samples after 1h mirror exposure. As can be seen in Fig. 1l, these fish showed a large spectrum of behaviour and there was a partial overlap of time spent interacting with the mirror between the groups. We thus decided to analyse two cohorts of fish from this experiment. We first evaluated transcriptomic differences between HAZ and LAZ that showed similar mirror aggression levels (cohort 1). We then evaluated transcriptomic differences between the behavioural extremes of the two groups, the most aggressive HAZ versus the least aggressive LAZ of the experiment (cohort 2).

Fish from the first cohort did not show any significant difference in behaviour during MIA (Additional file 1: Fig. S3), but did show strong transcriptomic differences. PCA again revealed a clear separation between HAZ and LAZ along PC1 of the plot, but no separate clusters for male and female fish from the respective lines (Fig. 3d). Like in F4 fish not exposed to the mirror, hierarchical clustering revealed a clear separation of HAZ and LAZ samples for both male and female fish (Fig. 3e, f), but differential expression analysis revealed considerably more DEGs. Specifically, female high aggression zebrafish (HAZf) had 243 protein-coding DEGs (110 upregulated and 133 downregulated) compared to female low aggression zebrafish (LAZf) from this cohort (Fig. 3g) and male high aggression zebrafish (HAZm) had 209 protein-coding DEGs (97 upregulated and 112 downregulated) compared to male low aggression zebrafish (LAZm) (Fig. 3h). In contrast, only 4 genes were differentially expressed between HAZf and HAZm and 12 genes between LAZf and LAZm indicating only minor sex-specific effects within the groups (Additional file 1: Fig. S4).

**Fig. 4:**
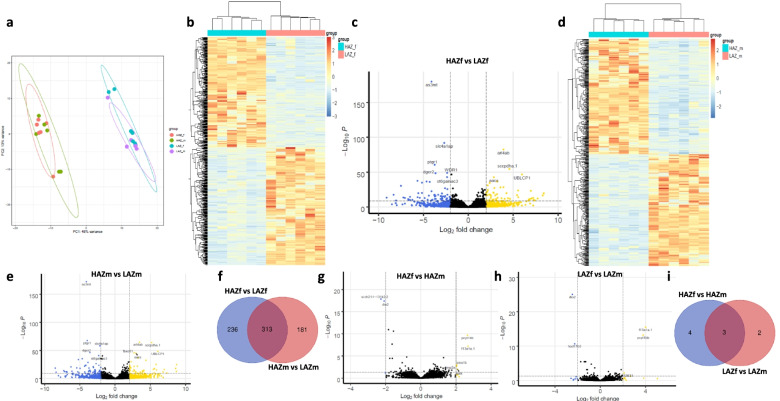
Neurotranscriptomic differences of the most aggressive male and female high aggression zebrafish (HAZ) compared to the least aggressive low aggression zebrafish (LAZ). **a** Principal component analysis plot of the top 200 most variable genes after differential expression analysis. *n* = 6/group. **b** Heatmap of differentially expressed genes (DEGs; padj < 0.05 and LFC > |2|) between female HAZ (HAZf) and female LAZ (LAZf) of cohort 2. Hierarchical clustering of samples and genes reveals large differences between HAZ and LAZ, but similar transcriptional patterns within aggression subgroups. *n* = 6/group. **c** Volcano plot displaying DEGs between HAZf and LAZf. DEGs with the lowest adjusted *p* value (padj) are highlighted. *n* = 6/group. **d** Heatmap of differentially expressed genes (DEGs; padj < 0.05 and LFC > |2|) between male HAZ (HAZm) and male LAZ (LAZm) of cohort 2. Hierarchical clustering of samples and genes reveals large differences between HAZ and LAZ, but similar transcriptional patterns within aggression subgroups. *n* = 6/group. **e** Volcano plot displaying DEGs between HAZm and LAZm. DEGs with the lowest adjusted *p* value (padj) are highlighted. *n* = 6/group. **f** Venn diagram showing the overlap of DEGs between the HAZf vs. LAZf comparison and the HAZm vs. LAZm comparison. **g** Volcano plot displaying DEGs between HAZf and HAZm. DEGs are highlighted. *n* = 6/group. **h** Volcano plot displaying DEGs between LAZf and LAZm. DEGs are highlighted. *n* = 6/group. **i** Venn diagram showing the overlap of DEGs between the HAZf vs. HAZm comparison and the LAZf vs. LAZm comparison. Golden dots in Volcano plots indicate genes upregulated in HAZ more than log fold change 2, blue dots represent genes downregulated in HAZ more than LFC -2 and black dots represent genes not passing these thresholds. Source data and individual data values are available at the ebrains data repository, DOI: 10.25493/VTP5-8J9 and in Additional file 2

When analysing the behaviour of the most aggressive HAZ compared to the least aggressive LAZ during prolonged MIA (cohort 2), we detected extreme differences in mirror interaction between HAZ and LAZ (main effect: *F*_(1,20)_ = 110.487; *P* < 0.001) but no sex differences. HAZ spent on average 1117.95 s in the mirror zone during the testing period, whereas LAZ interacted with the mirror for an average of only 3.93 s with half of the LAZ fish not interacting with the mirror at all (Additional file 1: Fig. S5). These stark differences in behaviour between these HAZ and LAZ were accompanied by major transcriptomic differences in the brains of these fish. PCA revealed a clear separation of HAZ and LAZ along PC1 of the plot explaining 48% of the total variance. In contrast, male and female zebrafish did not form separate clusters (Fig. 4a). In line with this finding, differential expression analysis showed a large number of DEGs between aggression subgroups, but not between males and females. Specifically, female mirror fighters (HAZf) of this cohort had 549 protein-coding DEGs (266 upregulated and 283 downregulated) compared to the least aggressive female LAZ (LAZf), which exhibited no or very little mirror fighting behaviour (Fig. 4b, c). Hierarchical clustering of samples revealed a clear separation between HAZ and LAZ indicating similar transcriptional patterns within aggression subgroups (Fig. 4b). Similar to female animals, male mirror fighters (HAZm) of this cohort had 494 protein-coding DEGs compared to LAZm (251 upregulated and 243 downregulated; Fig. 4d, e) and hierarchical clustering of samples also revealed a clear separation between HAZ and LAZ (Fig. 4d).

Next, we analysed which genes were differentially expressed between HAZ and LAZ with and without mirror exposure and the degree of DEG overlap between conditions. When looking at the DEGs of cohort 2 animals that showed the largest expression changes between the groups, we found that the 10 most significant DEGs of female animals include both up- and downregulated genes in the HAZf group compared to LAZf (Fig. 4c, Additional file 1: Fig. S6). These DEGs are conserved in mammals and have been associated with diverse important biological functions such as arsenic metabolism (*arsenite methyltransferase*; *as3mt*), cell migration (*ADP-ribosylation factor-like 4ab*; *arl4ab*), immune system activity (*ptgr1*), actin cytoskeletal remodelling (*WD repeat domain 1*; *wdr1*), corticogenesis (*DiGeorge syndrome critical region gene 2*; *dgcr2*), protein dephosphorylation (*ubiquitin-like domain containing CTD phosphatase 1*; *ublcp1*), sialic acid metabolism (*ST6 (alpha-N-acetyl-neuraminyl-2,3-beta-galactosyl-1,3)-N-acetylgalactosaminide alpha-2,6-sialyltransferase 3*; *st6galnac3*) and ketone body metabolism (*acetoacetyl-CoA synthetase*; *aacs*) [26–32] as well as with unknown biological functions (*slc4a1ap*, *sccpdha.1*). The 10 most significant DEGs for male animals in this cohort are highlighted in Fig. 4e and shown in Additional file 1: Fig. S7. Strikingly, the most significant DEGs between conditions in both males and females is *as3mt* indicating an important role during mirror fights independent of sex. We also found a large degree of overlap in highly significantly changed DEGs in HAZm vs LAZm compared to HAZf vs LAZf, with *slc4a1ap*, *arl4ab*, *ptgr1*, *sccpdha.1*, *dgcr2*, *ublcp1*, *aacs* and *st6galnac3* appearing in both lists. In fact, most DEGs between HAZf and LAZf (313 genes) of this cohort are also differentially expressed between HAZm and LAZm and vice versa (Fig. 4f). This overlap is also very high when analysing, for example, the 25 most significant (21 differentially expressed in HAZf vs. LAZf and HAZm vs. LAZm) or the 100 most significant DEGs in both comparisons (71 differentially expressed in HAZf vs. LAZf and HAZm vs. LAZm) (Additional file 2). This suggests a strong common neurotranscriptional basis of mirror fighting in males and females in agreement with our behavioural data. In contrast, considerably fewer genes were differentially expressed between male and female zebrafish within the HAZ and LAZ groups of cohort 2. HAZf zebrafish had 7 DEGs (5 upregulated and 2 downregulated) compared to HAZm (Fig. 4g) and LAZf showed 5 DEGs (3 upregulated and 2 downregulated) compared to LAZm (Fig. 4h). Three of these DEGs (*coagulation factor XIII, A1 polypeptide a, tandem duplicate 1* (*f13a1a.1*), *iodothyronine deiodinase 2* and *phosphate cytidylyltransferase 1B, choline b*) were differentially expressed between both HAZf vs HAZm and LAZf vs LAZm (Fig. 4i). Taken together, this analysis suggests that there are neurotranscriptional sex differences, but these are minor compared to the differences detected between HAZ and LAZ and they are not detectable at the behavioural level.

**Fig. 5:**
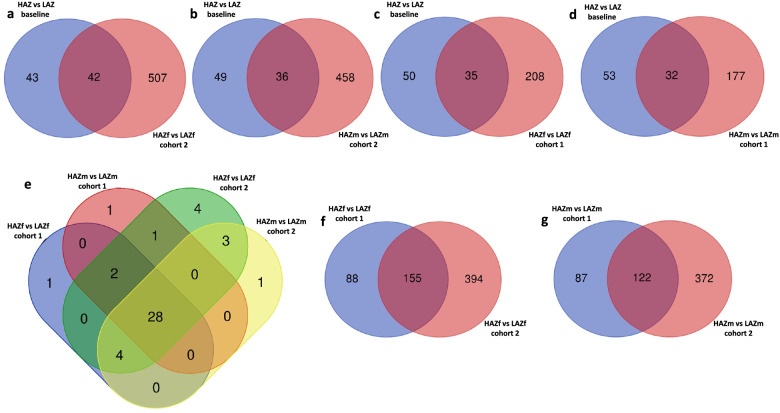
Overlap of differentially expressed genes (DEGs) between high aggression zebrafish (HAZ) and low aggression zebrafish (LAZ) at baseline and after mirror exposure. **a** Venn diagram showing the overlap of DEGs between the HAZ vs. LAZ comparison at baseline and the comparison of female cohort 2 fish. **b** Venn diagram showing the overlap of DEGs between the HAZ vs. LAZ comparison at baseline and the comparison of male cohort 2 fish. **c** Venn diagram showing the overlap of DEGs between the HAZ vs. LAZ comparison at baseline and the comparison of female cohort 1 fish. **d** Venn diagram showing the overlap of DEGs between the HAZ vs. LAZ comparison at baseline and the comparison of male cohort 1 fish. **e** Venn diagram depicting common DEGs between the comparisons of male and female cohort 1 and cohort 2 fish. **f** Venn diagram showing the overlap of DEGs between the comparison of female cohort 1 fish and the comparison of female cohort 2 fish. **g** Venn diagram showing the overlap of DEGs between the comparison of male cohort 1 fish and the comparison of male cohort 2 fish. For all comparisons, cohort 1 denotes HAZ and LAZ exposed to the mirror for 1h and displaying similar aggression levels, whereas cohort 2 denotes the comparison between the most aggressive HAZ during prolonged mirror exposure and the least aggressive LAZ. Source data and individual data values are available in Additional file 2

To disentangle which of the DEGs between HAZ and LAZ are related to selective breeding and which DEGs are related to mirror fighting, we analysed the DEG overlap between mirror-exposed individuals (cohorts 1 and 2) and fish not exposed to a mirror. These comparisons revealed an overlap of 32-42 DEGs (Fig. 5a–d) with a core set of 28 genes present in all comparisons (Fig. 5e). When subtracting genes that were already differentially expressed in the F0 generation (*ptgr1* and *lonrf1*), 26 genes remained, reflecting stable gene expression changes induced by selective breeding independent of mirror exposure (Additional file 3: Table 1). However, when considering the large amount of DEGs between cohort 1 HAZ and LAZ fish (males: 209 DEGs, females: 243 DEGs), which spent a similar amount of time interacting with the mirror (LAZ: 608.9 ± 108.2 s vs HAZ: 429.5 ± 91.58 s), it seems likely that selective breeding led to additional gene expression changes unmasked by mirror exposure. Finally, we investigated, which genes are induced by mirror exposure. For this, we subtracted the stably expressed genes induced by selective breeding mentioned above (Additional file 3: Table 1) and also the overlap of DEGs between mirror-exposed individuals (cohort 1 versus cohort 2 fish; Fig. 5f, g), reflecting additional genes induced by selective breeding, from the DEG lists of cohort 2. This results in two lists of genes of 395 and 372 DEGs (Additional file 4: Table 2, Additional file 5: Table 3) for females and males respectively, which are most likely induced by mirror fighting.

To validate the RNAseq findings, we performed RT-qPCR analysis of 6 DEGs between HAZm and LAZm across levels of significance, fold change and direction of change using an independent group of animals taken from the prolonged MIA experiment. There was a significant positive correlation between the RNAseq and qPCR expression changes (Pearson correlation coefficient of 0.869, *p* = 0.02) suggesting that our RNAseq data is reliable (Additional file 1: Fig. S8).

### Pathway analysis reveals an enrichment of immune-related pathways

**Fig. 6:**
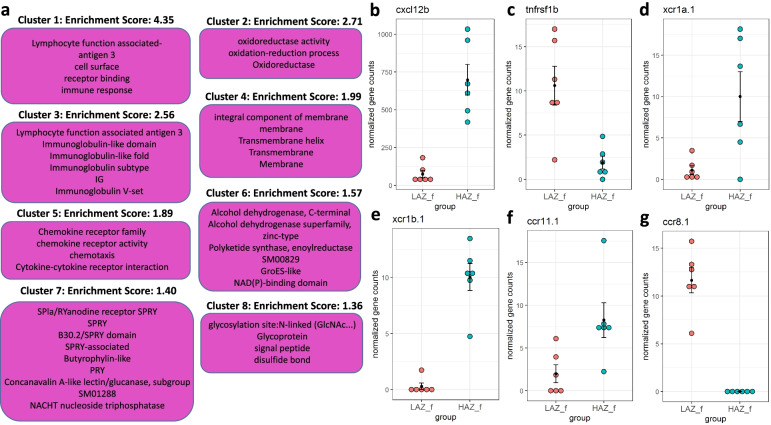
Enriched clusters and functional categories between the most aggressive female high aggression zebrafish (HAZf) and the least aggressive female low aggression zebrafish (LAZf). **a** Functional annotation clustering using DAVID pathway analysis revealed 8 significantly enriched clusters (enrichment score ≥ 1.3). Annotation terms related to each cluster are displayed in pink boxes. **b**–**g** DEGs with known important immunomodulatory functions present in one or more of these enriched clusters include **b** *chemokine (C-X-C motif) ligand 12b* (*cxcl12b*), **c** *tumour necrosis factor receptor superfamily member 1B* (*tnfrsf1b*), **d** *chemokine (C motif) receptor 1a, duplicate 1* (*xcr1a.1*), **e** *chemokine (C motif) receptor 1b, duplicate 1* (*xcr1b.1*), **f** *chemokine (C-C motif) receptor 11.1*(*ccr11.1*) and **g** *chemokine (C-C motif) receptor 8.1* (*ccr8.1*). *n* = 6/group. Source data and individual data values are available in Additional file 2

To investigate biological pathways underlying the observed gene expression changes, we used DAVID pathway analysis and weighted gene expression analysis (WGCNA). For this, we focused on cohort 2 of the prolonged MIA experiment, which showed the largest differences in behaviour and also gene expression. We used DAVID pathway analysis [33] to classify DEGs into functional clusters revealing 8 significantly enriched annotation clusters (enrichment score ≥1.3) in HAZf animals compared to LAZf from this cohort (Fig. 6). The most enriched annotation cluster for the HAZf vs LAZf comparison (cluster 1, enrichment score: 4.35) contains the GO term “immune response” and the INTERPRO term “Lymphocyte function associated antigen 3” suggesting differences in immunity-related pathways between the two groups. This notion is supported by the significant enrichment of two other clusters (Fig. 6a) containing the immunoglobulin-related terms “Immunoglobulin-like domain”, “Immunoglobulin-like fold”, “Immunoglobulin subtype”, “Immunoglobulin V-set” and “IG” (cluster 3, enrichment score: 2.56) and the chemotaxis-related terms “Chemokine receptor family”, “chemokine receptor activity”, “chemotaxis” and “Cytokine-cytokine receptor interaction” (cluster 5, enrichment score: 1.89). DEGs with important immunomodulatory functions present in one or more of these enriched clusters include *chemokine (C-X-C motif) ligand 12b (cxcl12b), tumour necrosis factor receptor superfamily member 1B (tnfrsf1b)* and various chemokine receptors (*chemokine (C motif) receptor 1a, duplicate 1* (*xcr1a.1*), *chemokine (C motif) receptor 1b, duplicate 1* (*xcr1b.1*), *chemokine (C-C motif) receptor 11.1*(*ccr11.1*), *chemokine (C-C motif) receptor 8.1* (*ccr8.1*)). (Fig. 6b–g, Additional file 1: Table 4).

**Fig. 7:**
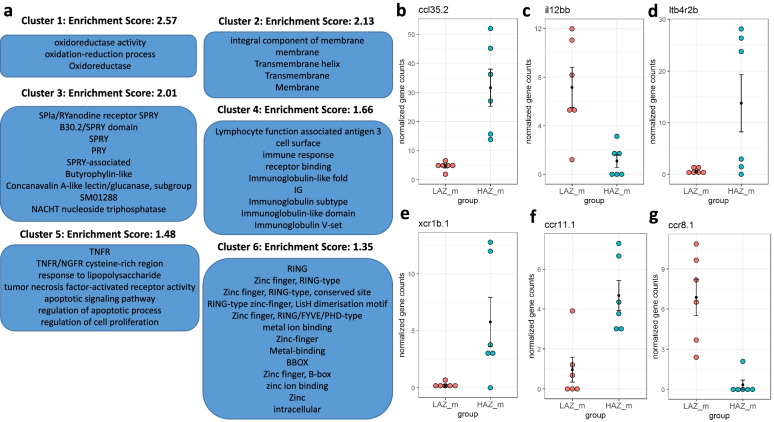
Enriched clusters and functional categories between the most aggressive male high aggression zebrafish (HAZm) and the least aggressive male low aggression zebrafish (LAZm). **a** Functional annotation clustering using DAVID pathway analysis revealed 5 significantly enriched clusters (enrichment score ≥ 1.3). Annotation terms related to each cluster are displayed in blue boxes. **b**-**g** DEGs with known important immunomodulatory functions present in one or more of these enriched clusters include **b** *chemokine (C-C motif) ligand 35 duplicate 2* (*ccl35.2*), **c** *interleukin 12B, c* (*il12bc*), **d** *leukotriene B4 receptor 2b* (*ltb4r2b*), **e** *chemokine (C motif) receptor 1b, duplicate 1* (*xcr1b.1*), **f** *chemokine (C-C motif) receptor 11.1* (*ccr11.1*) and **g** *chemokine (C-C motif) receptor 8.1* (*ccr8.1*). *n* = 6/group. Source data and individual data values are available in Additional file 2

Similarly, we detected an enriched cluster containing the immunoglobulin-related terms “immune response”, “Immunoglobulin-like fold”, “IG”, “Immunoglobulin subtype”, “Immunoglobulin-like domain”, “Immunoglobulin V-set” and “Lymphocyte function associated antigen 3” in male animals when comparing HAZm with LAZm (cluster 4, enrichment score: 1.66, Fig. 7a). In addition, this comparison showed another enriched cluster (cluster 5, enrichment score: 1.48) related to cytokine action containing the terms “TNFR” and “TNFR/NGFR cysteine-rich region”, “response to lipopolysaccharide” and “tumor necrosis factor-activated receptor activity”. DEGs in these enriched clusters with known important immunomodulatory functions include *chemokine (C-C motif) ligand 35 duplicate 2* (*ccl35.2*), *interleukin 12B, c* (*il12bc*), *leukotriene B4 receptor 2b* (*ltb4r2b*) and chemokine receptors (*xcr1b.1*, *ccr11.1*, *ccr8.1*) (Fig. 7b–g, Additional file 1: Table 5)

As well as enrichment of immune system-related processes, both comparisons (HAZf vs LAZf and HAZm vs LAZm) revealed an enriched cluster (enrichment score: 2.71 and 2.57, respectively) containing the GO terms “oxidoreductase activity”, “oxidation-reduction process” and the UniProt term “Oxidoreductase” (Figs. 6a and 7a). Most DEGs related to these terms were found when comparing both HAZf vs LAZf and HAZm vs LAZm (Additional file 1: Table 6). DEGs within these clusters encompass enzymes with a number of diverse biological functions such as nitric oxide synthesis (*nitric oxide synthase 2a, inducible*, *nitric oxide synthase 2b, inducible*), prostaglandin metabolism (*ptgr1*, *prostaglandin reductase 2*, *LOC566996*, *prostaglandin-endoperoxide synthase 2b*, *15-hydroxyprostaglandin dehydrogenase*) or steroid hormone metabolism (*cytochrome P450, family 11, subfamily A, polypeptide 1*, *cytochrome P450, family 2, subfamily AA, polypeptide 8*, *cytochrome P450, family 2, subfamily K, polypeptide16*, *cytochrome P450, family 3, subfamily C, polypeptide 4*, *hydroxysteroid (17-beta) dehydrogenase 2*) (Additional file 1: Table 6).

Finally, we detected the enrichment of a cluster containing the membrane-associated GO terms “integral component of membrane” and “membrane” as well as the UniProt terms “Transmembrane helix”, “Transmembrane” and “Membrane” (enrichment score: 1.99 and 2.13, respectively) in both HAZf vs LAZf and HAZm vs LAZm; Figs. 6a and 7a). More than 100 DEGs are part of this cluster in both HAZf vs LAZf and HAZm vs LAZm, with a large overlap again (Additional file 1: Table 7). Among the annotated DEGs within this cluster, we found a number of interesting genes encoding, for example, receptor proteins (e.g. *adrenoceptor alpha 1Bb (adra1bb)* and *corticotropin releasing hormone receptor 2 (crhr2)*), tight junction proteins (e.g. *claudin 11b (cldn11b)*) and cell adhesion proteins (e.g. *CD164 molecule, sialomucin*, *selectin E (cd164)*). Moreover, in line with the findings described above, many of the DEGs from these clusters have reported immunological functions including *leukotriene C4 synthase (ltc4s)*, *toll-like receptor 4b, duplicate a (tlr4ba)* or *tnfrsf1b* (Additional file 1: Table 7).

As expected, due to the low number of DEGs, pathway analysis did not show any enriched pathways for HAZf vs HAZm and LAZf vs LAZm.

### Weighted gene co-expression network analysis reveals unique gene modules associated with high and low mirror aggression

**Fig. 8:**
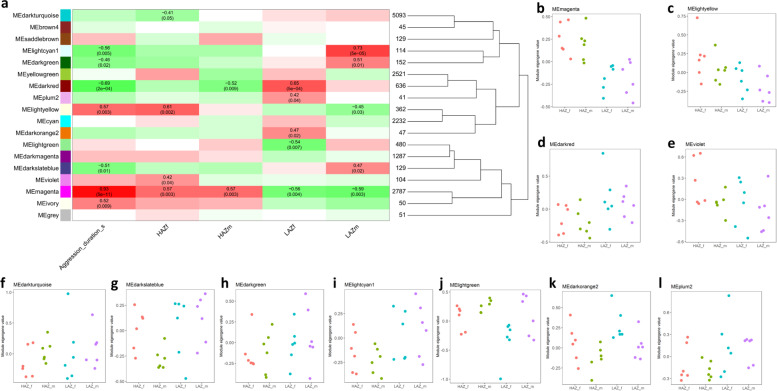
Weighted Gene Coexpression Network Analysis (WGCNA) of the most aggressive high aggression zebrafish (HAZ) compared to the least aggressive low aggression zebrafish (LAZ). **a** Correlations between gene coexpression modules identified by WGCNA and experimental group (male and female high aggression zebrafish (HAZm and HAZf), male and female low aggression zebrafish (LAZm and LAZf) as well as aggression duration. The colours of the boxes are scaled with the value of the correlation coefficient ranging from − 1 (green) to 1 (red). The *p* value of significant correlations and the respective correlation coefficient are shown in the Figure. **b**–**l** Eigengene values of samples separated by group (HAZf, HAZm, LAZf and LAZm) for gene modules significantly associated to one or more of the experimental groups or aggression duration. *n* = 6/group. **b** Gene coexpression module magenta, **c** gene coexpression module light yellow, **d** gene coexpression module dark red, **e** gene coexpression module violet, **f** gene coexpression module dark turquoise, **g** gene coexpression module dark slate blue, **h** gene coexpression module dark green, **i** gene coexpression module light cyan1, **j** gene coexpression module light green, **k** gene coexpression module dark orange2 and **l** gene coexpression module plum2. Source data and individual data values are available in Additional file 2

Weighted gene co-expression network analysis (WGCNA) was used to identify modules of highly correlated genes that are significantly associated with aggression duration or experimental group. Similar to DAVID pathway analysis, we decided to use the differentially expressed genes of cohort 2 for this analysis and were able to identify 18 co-expression gene modules containing between 41 and 5093 genes (Fig. 8a). To evaluate which of these gene modules are significantly associated with experimental groups or mirror interaction, associations between the eigengene values of each module (weighted average module expression profile) and experimental group or mirror interaction were determined yielding 12 gene co-expression modules that were significantly associated with aggression duration and/or the experimental groups (HAZm, HAZf, LAZm and LAZf). Only one of these gene modules (magenta) was significantly associated with aggression duration and all of the experimental groups. This module is enriched in genes related to a number of processes including cell projections, regulation of neurotransmitter levels, oxidative stress, oxidoreductase activity and lipid metabolism (Additional file 6: Table 8) and was positively correlated with aggression duration, HAZf and HAZm, but negatively correlated with LAZf and LAZm (Fig. 8a, b). Two other gene modules showed a similar association pattern to module magenta; that is in an opposite manner between HAZ and LAZ subgroups. Module light yellow (Fig. 8c), which is enriched in genes related to multicellular signalling, transmembrane transporter activity and the MAPK signalling pathway was positively correlated with HAZf, but negatively correlated with LAZm. In contrast, gene module dark red (Fig. 8d; enriched in genes related to cation transmembrane transporter activity, cell differentiation and the hedgehog signalling pathway) was positively correlated with LAZf, but negatively correlated with HAZm (Fig. 8a–d; Additional file 6: Table 8). Most experimental groups were also characterized by a significant association with at least one unique gene module that was not associated with the other experimental groups, suggesting that each experimental condition has a unique neurotranscriptomic state. Specifically, HAZf was characterized by a positive correlation with the violet module (Fig. 8e; enriched in genes related to regulation of gene expression, transcription factor binding and arginine and proline metabolism) and a negative correlation with the dark turquoise module (Fig. 8f; enriched in genes related to RNA processing, mitochondrial function, oxidoreductase activity and protein modification). Moreover, LAZm and LAZf were characterized by a significant association with three unique gene modules each. Module dark slate blue (enriched in genes related to ribonucleoprotein complex biogenesis, calmodulin binding and amino sugar and nucleotide sugar metabolism), module dark green (enriched in genes related to response to external stimulus, photo receptor activity, tryptophan and purine metabolism) and module light cyan1 (enriched in genes related to protein alkylation, oxidoreductase activity and beta-Alanine metabolism) were positively correlated with LAZm (Fig. 8g–i). Module light green (enriched in genes related to peripheral nervous system development, protein folding, lipid transporter activity and histidine metabolism) was negatively correlated with LAZf (Fig. 8j) and the modules dark orange2 (enriched in genes related to vesicle organization, endocytosis and autophagy) and plum2 (enriched in genes related to second-messenger-mediated signalling) were positively correlated with LAZf (Fig. 8k, l; Additional file 7: Table 9).

Aggression duration was significantly associated with seven gene modules. Gene modules ivory (enriched in genes related to lipid binding, serotonin receptor activity and tyrosine metabolism), magenta and light yellow were positively correlated with mirror interaction, whereas gene modules dark slate blue, dark red, dark green and light cyan1 were negatively correlated with this behaviour (Fig. 8a). The direction of correlations (positive or negative) shows high similarity for HAZf and HAZm, but not with the LAZ groups. This is most obvious for module magenta that shows a very strong positive correlation with aggression duration (*r*_*p*_ = 0.93), moderate positive correlations with HAZm (*r*_*p*_ = 0.57) and HAZf (*r*_*p*_ = 0.57), but also moderate negative correlations with LAZm (*r*_*p*_ = − 0.59) and LAZf (*r*_*p*_ = − 0.56). Given that HAZ were very aggressive against their mirror image and LAZ were not, this suggests that the genes within this module are induced by mirror aggression or are particularly important to determine the mirror aggression phenotype of zebrafish. A comparison of these genes with the differential expression analysis result reveals that 42.6% of the DEGs (234 genes) between HAZf and LAZf and 49.8% of the DEGs (246 genes) between HAZm and LAZm are part of this module (Additional file 7: Table 9, Additional file 8: Table 10) showing good overlap between the two different analysis methods used (DESeq2 and WGCNA). Strikingly, all of the 10 most significant DEGs from DESeq2 differential expression analysis are part of module magenta. In addition, 85 of the 100 most significant DEGs between HAZm and LAZm and 90 of the 100 most significant DEGs between HAZf and LAZf are part of module magenta as well. Two other modules (dark red and light yellow) were significantly correlated with aggression duration as well as two of the experimental groups. Module dark red, which is negatively correlated with aggression duration (*r*_*p*_ = − 0.69) and HAZm (*r*_*p*_ = − 0.52), but positively correlated with LAZf (*r*_*p*_ = 0.65) might reflect genes repressed by mirror aggression and module light yellow, which positively correlated with aggression duration (*r*_*p*_ = 0.57) and HAZf (*r*_*p*_ = 0.61), but negatively correlated with LAZm (*r*_*p*_ = − 0.45) might reflect another set of genes induced by mirror aggression. However, for these two modules, the overlap between the genes in each module and the DEGs that differ between HAZf and LAZf or HAZm and LAZm is much lower. Seventeen DEGs between HAZm and LAZm were also found in module dark red and 8 DEGs were also found in module light yellow. A similar overlap was found for DEGs between HAZf and LAZf, with 28 DEGs also appearing in module dark red and 15 DEGs also appearing in module light yellow.

### HAZ and LAZ display morphometric differences

**Fig. 9:**
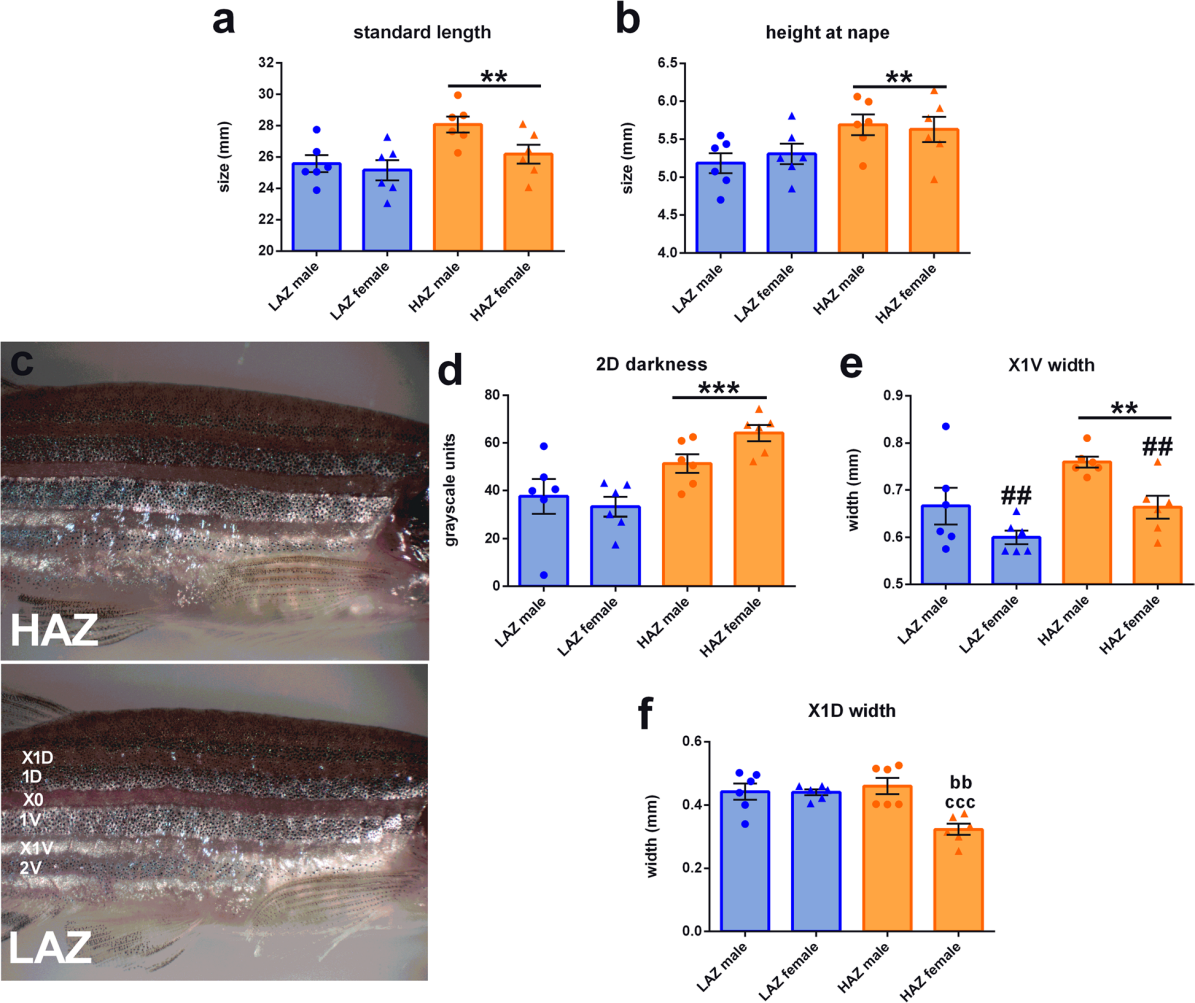
Morphological differences between high aggression zebrafish (HAZ) and low aggression zebrafish (LAZ). **a** Standard length and **b** height at nape measurements of male and female HAZ and LAZ exposed to the mirror-induced aggression setup for 1h and used for RNAseq (cohort 2). **c** Representative stripe pattern images of used HAZ and LAZ. **d** Stripe colouration of 2D stripes as assessed by grayscale measurements of stereomicroscopic images. (**e**, **f** Width of X1V and X1D interstripes. Two-way ANOVA followed by Tukey post hoc test in the case of a significant interaction term. *n* = 6/group. ***, *P*<0.001; **, *P*<0.01 main effect HAZ vs. LAZ; bb, *P*<0.01 vs. LAZ male; ccc, *P*<0.001 vs. HAZ male. Data are presented as mean ± SEM. Source data and individual data values are available in Additional file 2

Some species of fish display dominance or aggression by changing their external appearance or displaying certain body features [34]. Recent evidence suggests a transient darkening of zebrafish during dyadic fights [35], but it is currently unclear if mirror fighting and selection for aggressive traits leads to morphometric changes in zebrafish. Thus, we investigated whether the observed changes in mirror aggression, anxiety-like behaviour and gene expression are accompanied by changes in body appearance. Stereomicroscopic images of the fish used for RNAseq revealed that aggressive HAZ are slightly bigger than non-aggressive LAZ independent of sex as assessed by standard length (main effect: *F*_(1,20)_ = 9.269; *p* = 0.006; Fig. 9a) and height at nape (main effect: *F*_(1,20)_ = 8.371; *p* = 0.009; Fig. 9b). Body coloration as well as stripe and interstripe width was largely similar for HAZ and LAZ (Fig. 9c; Additional file 1: Fig. S9), but HAZ had darker 2D stripes (main effect: *F*_(1,20)_ = 20.508; *p* < 0.001; Fig. 9d), larger X1V interstripes independently of sex (Fig. 9e; main effect: *F*_(1,20)_ = 10.195; *p* = 0.005) and female HAZ had smaller X1D interstripes than male HAZ and female LAZ (Fig. 9f; sex X genotype interaction: *F*_(1,20)_ = 10.674; *p* = 0.004). In contrast, sex determined the width of several stripes and interstripes with females displaying larger 1V (sex main effect: *F*_(1,20)_ = 4.956; *p* 0.038) and 2V (sex main effect: *F*_(1,20)_ = 28.687; *p* <0.001) stripes than males independently of mirror aggression phenotype, but smaller X0 (sex main effect: *F*_(1,20)_ = 43.685; *p* < 0.001) and X1V (sex main effect: *F*_(1,20)_ = 10.827; *p* = 0.004) interstripes (Additional file 1: Fig. S9a–c; Fig. 9e). The size of the tail- and anal fin did not differ between HAZ and LAZ (Additional file 1: Fig. S9d, e), but female zebrafish had smaller anal fins than males independent of mirror interaction (sex main effect: *F*_(1,20)_ = 15.313; *p* < 0.001). Whole body coloration and 1D darkness was similar in all groups (Additional file 1: Fig. S9f, g), but we observed sex main effects in the colour of the 1V and 2V stripes (1V: *F*_(1,20)_ = 5.002; *p* = 0.037 and 2V: *F*_(1,20)_ = 6.806; *p* = 0.017), likely due to the clear differences in body appearance between males and females (Additional file 1: Fig. S9h, i).

## Discussion

What determines an animal’s aggressive interaction with its own mirror image? Exposing different species to a mirror has revealed that mirror aggression is a common behaviour [36–38]. However, the amount of time spent mirror fighting varies between individuals of the same species suggesting that both intrinsic and extrinsic factors influence its expression. Here we use zebrafish to demonstrate that mirror aggression has a strong genetic component. Selective breeding of high and low mirror aggression zebrafish showed that the mirror aggression phenotype is heritable over multiple generations, and RNAseq analysis revealed that zebrafish which display high mirror aggression levels show large transcriptomic differences in the brain compared to fish that do not interact with their mirror image.

Zebrafish are a social species and form shoals with hierarchies that can provide insights into the dynamics of group interactions [39]. However, when two zebrafish are placed together in a tank they often start to fight, and will eventually establish a dominance/subordinate relationship. Dominant zebrafish tend to be larger, exhibit more aggression and claim more territory within an aquarium [40–42]. Zebrafish also display signs of aggression towards their own mirror image, and mirror stimulation has been used as an alternative to dyadic fights in several studies [11, 15, 40, 43]. The selection strategy that we used in this study was very effective given that the offspring of HAZ showed higher mirror aggression levels compared to the offspring of LAZ in all generations investigated with the exception of juvenile F2 fish. This suggests an important genetic contribution to this behaviour in line with a previous heritability index estimation of 0.36 in zebrafish [13]. An interesting question for future studies is whether HAZ and LAZ are also more or less aggressive towards conspecifics, respectively. Previous studies using candidate gene knockout models revealed divergent results regarding this matter. For example *nos1*^*-/-*^ zebrafish are less aggressive in both mirror aggression and dyadic fights, but *fgfr1a*^*-/-*^ zebrafish are more aggressive in the mirror test but not in dyadic fights compared to respective wild-type animals [17, 44] indicating that different genes may regulate these two forms of zebrafish aggression. We also observed a correlation between high aggression and low anxiety levels in aggressive HAZ from the F3 generation onwards suggesting that HAZ might be better described as high aggression/low anxiety zebrafish and LAZ as low aggression/high anxiety zebrafish. A similar relationship between aggression and anxiety has been described in mice (e.g. tryptophan hydroxylase 2 knockout mice [45]), while studies in other rodents report the opposite phenotype (high aggression and high anxiety [46];). This apparent discrepancy might be explained by multiple different genes that affect aggression and anxiety, with a subset that influence both behaviours. Alternatively, different subtypes of aggression and anxiety may have been compared in these studies.

We have previously shown that mirror aggression increases expression of the neuronal activation marker ribosomal protein S6 (rpS6) [18]. We observed activation of multiple areas of the zebrafish brain including homologues of the mammalian amygdala and hippocampus, the lateral and medial zone of the dorsal telencephalic area. The dorsal and ventral nuclei of the ventral telencephalic area, the anterior parvocellular preoptic area, the lateral hypothalamus, caudal and ventral zones of the periventricular hypothalamus and the area around the posterior recess of the diencephalic ventricle were also activated. These changes were genotype-dependent, since zebrafish lacking *histamine H3 receptor* function had higher aggression-induced activation of *rpS6* in the medial zone of the dorsal telencephalic area and the area around the posterior recess of the diencephalic ventricle, but lower activation of the ventral nuclei of the ventral telencephalic area following a mirror test [18]. This supports the concept of a genetic influence on the areas of the brain activated by aggression. In a previous study Oliveira et al. [47, 48] reported significant differences in the brain transcriptome of fish experiencing a dyadic fight (winners and losers) compared to socially isolated fish, but no change in transcriptomic activation between mirror fighters and the other experimental groups. This is in stark contrast to the data presented herein, where we found large differences between mirror-exposed individuals. However, the different study designs (selective breeding vs. characterization of wild-type fish) and methodologies used (RNAseq vs. microarray) as well as the larger number of biological replicates in this study may explain this discrepancy. Interestingly, meta-analysis of the data from Oliveira et al. detected 70 DEGs in the brain when comparing zebrafish experiencing a fight (including mirror fighters) and isolated fish [21], which is in agreement with the data that we present here. Intriguingly, alterations in mitogen-activated protein kinases (MAPK) signalling was identified in all these studies suggesting that this may be an important signalling pathway to control aggression [15, 21, 47].

Using our approach, we showed that both selective breeding and mirror aggression induce changes to the brain transcriptome. Comparison of the baseline transcriptome between our founder fish (F0 generation) and fish selectively bred for high and low mirror aggression phenotype over 4 generations (F4 generation) revealed 85 DEGs likely induced by selective breeding. Twenty-six out of these 85 genes are also differentially expressed in F4 HAZ and LAZ after stimulus (mirror) exposure suggesting that these are stable gene expression changes between the two lines possibly regulating the propensity to interact with the mirror. In addition, we also uncovered numerous DEGs that are likely induced by mirror interaction independently of selective breeding. Comparison of the most aggressive mirror fighters with minimally or not aggressive fish revealed around 500 genes as differentially expressed. When correcting this list for the stable gene expression changes and additional genes possibly related to selective breeding, around 380 genes remain that are likely induced by mirror aggression. Among the DEGs uncovered in this study, *as3mt* stands out as being the most significant DEG between highly aggressive HAZ and minimally mirror-interacting LAZ as well as being one of the genes differentially expressed between HAZ and LAZ at baseline. *as3mt* codes for a methyltransferase that is important for arsenic detoxification and is ubiquitously expressed in zebrafish tissues including the brain [49]. Although the function of this enzyme in the zebrafish brain is unknown, polymorphisms in the human orthologue, *AS3MT*, have been linked to schizophrenia [50] and altered brain activation during a memory task [51] indicating a role in neural plasticity. Another interesting hit among the most significant DEGs is *dgcr2* encoding a putative adhesion receptor protein. Similar to *as3mt*, the human orthologue of this gene has been linked to schizophrenia [52]. *Dgcr2* knock-out or knockdown mice show substantial locomotor deficits, altered neuronal migration and impaired Purkinje cell function [29, 53]. We have previously suggested that Purkinje cell function may control zebrafish aggression [54] suggesting that this may represent an important node in a neural circuit that can control this behaviour. A third gene of interest is *aacs*, which encodes a ketone body-utilizing enzyme important for the synthesis of cholesterol and fatty acids. The enzyme is highly expressed in the brain and has been linked to neurogenesis and neuronal differentiation [55]. Interestingly, none of these candidates appears to modulate neurotransmitters that are usually associated with aggression such as serotonin or dopamine [56, 57]. Future studies should investigate their function in more detail, for example by creating knock-out lines and measuring changes to neurochemistry and behaviour.

DAVID pathway analysis and WGCNA revealed that a number of diverse pathways are altered between HAZ and LAZ. Perhaps most interestingly, genes and pathways related to immune function are linked to the mirror aggression phenotype. Emerging evidence indicates that neuroimmunological interactions are of paramount importance for brain function, behaviour and the course of psychiatric disorders [58]. In addition, changes to proinflammatory cytokine levels have been linked to aggression in both animals and humans [59]. Since aggression can be harmful for animals, animals with high levels of aggression may require stronger immune responses to recover from injury and to protect themselves from infection [59]. Anxiety has also been linked to changes within the immune system, which is interesting in the current context, given that HAZ are less anxious than LAZ. For example, a recent study in mice showed that IL-17a secreted from meningeal γδ17 T cells expressing high levels of chemokine receptor 6 regulates anxiety-like behaviour [60] and a recent meta-analysis found that maternal immune activation during pregnancy induces anxiety behaviour in offspring [61]. In line with these findings, LAZ and HAZ show differences in cytokine expression (*il12bb*, *tnfrsf1b*, *cxcl12b*, *ccl35.2* and *macrophage expressed 1, tandem duplicate 2*) chemokine receptor expression (*xcr1a.1*, *xcr1b.1*, *ccr11.1* and *ccr8.1*) and other genes related to immune system activity (*ptgr1*, *ltb4r2b*, *ltc4s*, *tlr4ba* and *toll-like receptor 8a*). Most of these genes are overexpressed in HAZ indicating that mirror aggression activates the immune system. For example, 3 out of 4 differentially expressed chemokine receptors (*xcr1a.1*, *xcr1b.1*, *ccr11.1*) and both chemokine receptor ligands (*cxcl12b*, *ccl35.2*) show higher expression in male and female HAZ samples compared to respective LAZ samples. *XCR1* is the human homologue of the *xcr1a.1* and *xcr1c.a* genes, which has been duplicated in zebrafish. In humans, it is expressed by a subset of dendritic cells and is important for dendritic-cell-mediated cytotoxic immune responses [62]. Also of note is the overexpression of the *cxcl12b* gene, an important factor for neutrophil migration in zebrafish [63] and the downregulation of *ptgr1*, an enzyme that inactivates the proinflammatory mediator leukotriene B4 and thus dampens inflammation [27].

Differences in behaviour between males and females are present throughout the animal kingdom. Regarding aggression, there is a large body of evidence suggesting sex differences in aggressive behaviour of many species with males often being more aggressive than females (e.g. [64, 65]). However, aggression is also a prominent behaviour in females, which still lacks detailed characterization at the neurobiological and genetic levels [66]. In fish, species-specific sex differences in aggressive behaviour have also been reported [67]. However, few studies have investigated the influence of sex on aggressive behaviour during mirror exposure systematically. Studies in diverse species including Siamese fighting fish [68], jumping spiders [69] and rainbow kribs [70] failed to detect sex differences in aggression, in line with our results. In agreement with the behavioural data, we also found only a small number of DEGs between male and female HAZ as well as male and female LAZ and no significantly enriched pathway. Interestingly, one of the few genes that were differentially expressed between males and females independently of mirror aggression phenotype is *f13a1a.1*. This gene has recently also been identified in another study characterizing transcriptomic differences in the brain of male and female zebrafish [71], but its function in the brain is currently unknown.

A potential explanation as to why some animals are aggressive against their own mirror image while others are not, might be differences in the images perceived by the animal looking into the mirror. Among fish, there are a number of examples whereby external body features such as black colour patches or melanic colour have been associated with aggression [34]. However, we did not find much evidence that aggression duration during mirror exposure depends on the body features of the fish under investigation. The size and appearance of fins and most stripes were similar between HAZ and LAZ with the exception of a bigger X1V interstripe and a darker 2D stripe in HAZ. In contrast, many more differences in morphology were observed between males and females, but this did not translate into differences in mirror aggression suggesting that the perceived mirror reflection of an individual is not the primary driver for this behaviour.

## Conclusions

The current study shows that selective breeding based on mirror aggression phenotype induces strong, heritable changes in behaviour and gene expression within the brain of zebrafish suggesting an important genetic basis of aggression in this species. Our transcriptomic analysis of fish selectively bred for high and low levels of mirror aggression revealed specific signatures induced by selective breeding and mirror aggression and thus provides a large and novel resource of candidate genes for future study. Many of these genes have not been characterized in detail and have the potential to help better understand the genetic basis of this behaviour or other forms of aggression.

## Methods

### Animal care and maintenance

Zebrafish were maintained at the University of Leicester using standard husbandry protocols and in accordance with institutional guidelines for animal welfare. The fish were maintained on a 14-h light/10-h dark cycle and fed twice a day. Experiments were carried out using AB wild-type zebrafish from the local breeding colony, which were selected based on their aggression levels before being inbred. All animals were kept at the same stocking density of 5-6 adult/fish per litre to ensure consistent environmental conditions. Animal experiments were approved by a local Animal Welfare and Ethical Review Body (AWERB) and conducted under the UK Home Office project and personal licences.

### Selective breeding strategy

Forty 4–7-month-old AB wild-type zebrafish (22 females, 18 males) were randomly selected from three different tanks in the local breeding colony (F0 generation) and tested for their mirror-induced aggression (MIA) behaviour. The three most aggressive males and females were selected and group-incrossed as were the three least aggressive males and females. These fish and their offspring were named high mirror aggression zebrafish (HAZ) and low mirror aggression zebrafish (LAZ), respectively. To maximize selection for aggressive traits over generations, the age-matched offspring of the F0 fish (F1 generation) were tested for MIA levels at 1 month of age [72]. The 20 most aggressive HAZ and the 20 least aggressive LAZ identified in this test were raised to adulthood and retested for aggression in the MIA setup at 3 months and on the following day also for anxiety in the novel tank diving test (NTD). The three most aggressive male and female HAZ were again incrossed as were the three least aggressive male and female LAZ to obtain a new generation (F2 generation). This strategy was repeated for two more generations to obtain a F4 generation of HAZ and LAZ that was used for further experiments.

### Mirror-induced aggression

The MIA paradigm was used to characterize juvenile (1 month old) and adult (3 months old) aggression levels as previously described. Juvenile mirror-induced aggression was measured using an automatic setup consisting of a soundproof cubicle and small tanks permitting 12 fish to be tested in parallel [72]. After 5 min habituation to the setup, mirror aggression was measured by removing an opaque barrier and permitting the fish to view the mirror. The behaviour of the fish was videotaped for 5 min and analysed automatically using Viewpoint software. Afterwards, the test animals were single-housed until analysis. Fish selected based on their aggression levels were raised to adulthood in groups of 20. Adult mirror aggression was quantified by placing fish in a narrow tank measuring 15 × 10 × 30 cm with a mirror placed outside the tank at an angle of 22.5° (Gerlai et al., 2000). Behaviour was recorded for 5 min as described in [15]. The fish’s movements and interaction with the mirror were recorded by a camera connected to FlyCap2 software. Aggressive acts against the mirror image were defined as biting and thrashing the tail fin. Aggression was scored manually by an experienced investigator blind to the experimental groups. After the MIA test adult zebrafish were single-housed until anxiety was tested on the next day.

### Novel Tank Diving (NTD)

The NTD test was used to measure anxiety-like behaviour [73]. For this, zebrafish were placed individually in a trapezoid tank (length × width × height; 19 × 10 × 7 cm) and filmed for 5 min. The behaviour of fish in the tank was videotracked using Ethovision XT12 software. The tank was divided horizontally into 3 equal parts for analysis. Fish that spent less time in the top compartment were considered more anxious [16].

### Open field test

The open tank test was used to evaluate locomotor activity. Individual zebrafish were placed in a large tank (37.5 × 21.5 × 15 cm; length × width × height) and their behaviour was recorded for 5 min. Ethovision XT12 software was used to analyse the swimming trajectory of the fish and to calculate the total distance moved, velocity, time spent immobile and angular velocity [16].

### Shoaling behaviour

Shoaling behaviour of LAZ and HAZ was evaluated in a group of 5 familiar fish (*n* = 9 shoals/group) by placing them into a large tank (37.5 × 21.5 × 15 cm; length × width × height) filled with aquarium water as previously described [18]. After 5 min of habituation, the fish were filmed from above for 20 min. Nearest neighbour distances (NND) and inter-individual distances (IID) were calculated using VpCore2 software (ViewPoint Life Sciences, Lyon, France).

### Social interaction

The visually-mediated social preference test was used to investigate social interactions as previously described [74]. Briefly, a test fish (LAZ or HAZ) was placed in the central compartment of the VMSP tank (length × width × height; 19 × 13.2 × 9.3 cm), which is surrounded by 4 smaller compartments (length × width × height; 9.2 × 6.5 × 9.3 cm). All walls of the tank are made of clear acrylic to enable fish to see each other and to facilitate video recording and tracking. One of the smaller compartments was then filled with 3 unfamiliar stimulus AB wild-type fish, while the other compartments remained empty. Time spent near the stimulus fish compartment (social interaction zone) during a 5-min test period was used as a readout for social behaviour.

### Novel object boldness

Novel object boldness was measured by placing LAZ or HAZ in a large tank (37.5 × 21.5 × 15 cm; length x width x height) containing an unfamiliar object as previously described [15]. The novel object, resembling a predator fish, was made by filling a 15-cm-long clear plastic tube with dark modelling clay. This object was suspended at one end of the tank, midway in the water column. The amount of time that a test fish spent within one fish body length near the novel object during a 5 min test was quantified using Ethovision XT12 software and considered as an index of boldness.

### RNAseq

To analyse the brain transcriptome of HAZ and LAZ, 30 fish each were exposed to the adult mirror-induced aggression setup for 1h. The fish were videotaped and Ethovision XT12 was used to quantify the time interacting with the mirror, distance travelled and time spent immobile. After this period, fish were immediately sacrificed in ice-cold water, body images were taken under a stereomicroscope to evaluate external body features and brains were collected. Out of these 30 animals, two cohorts of animals were selected for transcriptomic analysis. On the one hand, six animals/group displaying similar aggression levels were chosen (cohort 1), and on the other hand, the brains of the 6 most aggressive male and female HAZ as well as the 6 least aggressive male and female LAZ (cohort 2) were processed for RNAseq. Additionally, we processed the brains of HAZ and LAZ founders (F0 generation; *n*=3/group) and of F4 HAZ and LAZ not exposed to mirror (*n*=3/group). For this RNA was isolated using peqGOLD Trifast (Thermo Scientific) followed by DNase digestion (Ambion DNA-free™ DNA Removal Kit, Thermo Scientific) according to the manufacturer’s instructions as previously described (Reichmann et al. 2020). RNA quality was checked on a Bioanalyzer 2100 (Agilent, UK), which revealed that all samples had an RNA Integrity Number of at least 8.8 and thus were used for sequencing. Indexed libraries were prepared using the NEBNext® Single Cell/Low Input RNA Library Prep Kit for Illumina, according to the manufacturer’s protocol (New England Biolabs, UK). All libraries were then pooled at equipmolar concentration and sequenced by paired-end (2×150bp) sequencing on an Illumina Novoseq sequencer at Novogene Co., Cambridge, UK.

### Bioinformatics

The generated paired-end raw sequence data with 3.48 E+09 total number of reads (mean 7.25E+07 stdev 3.79E+07) was quality controlled and sequencing adapters as well as reads shorter than 50 base pairs were removed with Trim Galore! (Galaxy Version 0.4.3.1) to increase the mapping quality. We reached on average 42.9 (stdev 20.3) Million reads (min 14.2 and max 125.0) over all 48 samples after Trim Galore!. On average 67.5% (stdev 0.06%) of the reads could be successfully uniquely mapped with the RNAStar aligner (Galaxy Version 2.7.2b) [75] to the zebrafish reference genome GRCz11. Final transcript count data was generated with the HTSeq framework (Galaxy Version 0.9.1) for high throughput sequencing data [76] based on Ensemble release 99 gene annotation using standard settings. All analysis was conducted on a private Galaxy instance running on the MedBioNode cluster from the Medical University Graz. Further downstream analysis was conducted with the statistical program R version 3.6.3 within the free RStudio Desktop version. Differential gene expression analysis was performed with DESeq2 package version 1.26 [77] on the count table as output from HTSeq framework. DAVID Bioinformatics Resources 6.8 [33] was used for pathway enrichment analysis by clustering DEGs and associated biological annotation terms into functional groups. Enrichment score cutoff in DAVID was set to 1.3, which corresponds to a corrected *p* value of 0.05. We used Weighted Correlation Network Analysis (WGCNA) R package [78] to find clusters of coexpressed genes following the step-by-step tutorial of the developers. As input 50% of most variable normalized genes were selected and soft thresholding parameter 8 was applied to which co-expression similarity is raised to calculate adjacency leaving the rest of the calculation with standard parameters.

### qPCR validation of RNAseq data

To validate RNAseq findings, brains from a separate cohort of HAZ and LAZ animals exposed to MIA for 1h but not used for RNAseq were processed for qPCR (*n* = 6/group). As described above, RNA was extracted using peqGOLD Trifast (Thermo Scientific) followed by DNase digestion (Ambion DNA-free™ DNA Removal Kit, Thermo Scientific) and then reverse-transcribed using the RevertAid First Strand cDNA Synthesis Kit (Thermo Scientific) according to manufacturer instructions. For relative quantification of mRNA levels, qPCR was performed on a CFX384 Touch™ Real-Time PCR Detection System (BioRad, Vienna, Austria) using SsoAdvanced Universal SYBR Green Supermix (Biorad) [18]. Six protein-coding genes (*as3mt*, *neuropeptide Y receptor Y8b*, *cd164*, *ptgr1*, *frizzled class receptor 4* and *collagen*, *type XII*, *alpha 1b*) were semi-randomly selected across levels of significance, fold change and direction of expression change during the RNAseq experiment and primers for these genes were designed with Primer BLAST (Additional file 1: Table 11). Correct sequence amplification was confirmed by Sanger sequencing. All samples were measured as triplicates. *actin beta1* and *ribosomal protein L13a*, which were not differentially expressed in the RNAseq dataset, were used as reference genes for quantification of target gene expression. Quantitative measurements of target gene levels relative to LAZm were performed with the ΔΔ Cq method using the mean value of the LAZm group as the calibrator. Group differences were expressed as fold changes and then converted to log2 fold change to enable comparison with the RNAseq dataset.

### External body features

Body characteristics of HAZ and LAZ were compared by measuring standard length and height at nape as well as by comparing stripe and fin characteristics. For this, fish were euthanized in ice water directly after a 1h MIA exposure and immediately photographed in a petri dish filled with ice water. Images were acquired on a GXM L3200B light microscope (GT Vision, Stansfield, UK) and Fiji software was used for image processing and analysis. The width of stripes (1D, 1V, 2V) and interstripes (X1D, X0, X1V) were measured 5 times each at randomly picked, evenly spaced locations between the gills and tail fin base of each fish and the mean thereof was used for statistical analysis. The standard length was measured from the snout to the tail fin base and height at nape was measured from ventral to dorsal, immediately posterior to the head, perpendicular to the axis defined by standard length [79]. The tail and anal fin area was determined with the polygon tool in Fiji. To analyse the melanic colouration of stripes and the whole fish body, mean grayscale values were obtained. Stripe grayscale was measured 6–10 times with 0.0625 mm^2^ (2D, 2V) or 0,25 mm^2^ (1D, 1V) boxes depending on stripe thickness. Mean values were used for statistics.

### Statistics

Statistical analysis was performed using IBM SPSS Statistics 26 (IBM, Armonk, USA) and GraphPad Prism 6 software packages (Graphpad Software Inc, San Diego, USA) except for RNAseq bioinformatics analysis, which is described in detail above. Group differences between the two groups were assessed by the Mann-Whitney *U* test. Group differences for analysis with 2 factors (mirror aggression phenotype and sex) were assessed by two-way ANOVA followed by main effect analysis or Tukey post hoc test in case of a significant interaction term. The Pearson correlation coefficient was used to assess correlations between aggression duration and time spent in the top zone of the novel tank diving test. *P* values < 0.05 were considered statistically significant.

## Supplementary Information

**Additional file 1: Fig. S1.** High aggression zebrafish (HAZ) and low aggression zebrafish (LAZ) show similar locomotor activity, social behaviour and boldness. **Fig. S2.** Neurotranscriptomic differences of founder (F0) high aggression zebrafish (HAZ) and low aggression zebrafish (LAZ). **Fig. S3.** Behaviour of cohort 1 high aggression zebrafish (HAZ) and low aggression zebrafish (LAZ) during prolonged mirror exposure. **Fig. S4.** Sex-specific neurotranscriptomic differences between mirror-exposed high aggression zebrafish (HAZ) and low aggression zebrafish (LAZ). **Fig. S5.** Behaviour of the most aggressive high aggression zebrafish (HAZ) and least aggressive low aggression zebrafish (LAZ) during prolonged mirror exposure (cohort 2). **Fig. S6.** Ten most significant differentially expressed genes (DEGs) between female high aggression zebrafish (HAZf) and low aggression zebrafish (LAZf). **Fig. S7.** Ten most significant differentially expressed genes between male high aggression zebrafish (HAZm) and low aggression zebrafish (LAZm). **Fig. S8.** qPCR validation of RNAseq findings. **Fig. S9.** Morphological differences between male and female zebrafish independently of mirror aggression phenotype. **Table 4.** Differentially expressed genes between HAZf and LAZf associated with immune system related pathway terms obtained by DAVID pathway analysis. **Table 5.** Differentially expressed genes between HAZm and LAZm associated with immune system related pathway terms obtained by DAVID pathway analysis. **Table 6.** Differentially expressed genes involved in oxidation reduction processes for HAZf vs. LAZf and HAZm vs. LAZm obtained by DAVID pathway analysis. **Table 7.** Differentially expressed genes involved in membrane-related processes for HAZf vs. LAZf and HAZm vs. LAZm obtained by DAVID pathway analysis. **Table 11.** Primers used for real-time RT PCR to validate RNAseq findings.

**Additional file 2.** Supporting data underlying Fig. 1-9 and Fig. S1-9.

**Additional file 3: Table 1.** List of differentially expressed genes between F4 HAZ and LAZ independent of mirror exposure.

**Additional file 4: Table 2.** List of differentially expressed genes between female F4 HAZ and LAZ induced by mirror aggression.

**Additional file 5: Table 3.** List of differentially expressed genes between male F4 HAZ and LAZ induced by mirror aggression.

**Additional file 6: Table 8.** GO and KEGG pathway terms associated with gene coexpression modules obtained by WGCNA.

**Additional file 7: Table 9.** Comparison of genes from WGCNA gene module magenta with DEGs between HAZf and. LAZf identified by DEseq2.

**Additional file 8: Table 10.** Comparison of genes from WGCNA gene module magenta with DEGs between HAZm and. LAZm identified by DEseq2.

## Data Availability

All data generated or analysed during this study are included in this published article, its supplementary information files and publicly available repositories. RNAseq data generated and analysed during the current study are available in the ebrains data repository, DOI: 10.25493/VTP5-8J9. Other source data and individual data values are available in Additional file 2

## Abbreviations

aacs: Acetoacetyl-CoA synthetase
adra1bb: Adrenoceptor alpha 1Bb
arl4ab: ADP-ribosylation factor-like 4ab
as3mt: Arsenite methyltransferase
ccl35.2: Chemokine (C-C motif) ligand 35 duplicate 2
ccr8.1: Chemokine (C-C motif) receptor 8.1
ccr11.1: Chemokine (C-C motif) receptor 11.1
cd164: CD164 molecule, sialomucin, selectin E
cxcl12b: (C-X-C motif) ligand 12b
cldn11b: Claudin 11b
crhr2: Corticotropin-releasing hormone receptor 2
dgcr2: DiGeorge syndrome critical region gene 2
ednraa: Endothelin receptor type Aa
DEGs: Differentially expressed genes
f13a1a.1: Coagulation factor XIII, A1 polypeptide a, tandem duplicate 1
fgfr1a: Fibroblast growth factor receptor 1a
HAZ and LAZ: High and low mirror aggression zebrafish
HAZf: Female high aggression zebrafish
HAZm: Male high aggression zebrafish
hrh3: Histamine receptor H3
IID: Inter-individual distances
il12bc: Interleukin 12B, c
LAZf: Female low aggression zebrafish
LAZm: Male low aggression zebrafish
lonrf1: LON peptidase N-terminal domain and ring finger 1
ltb4r2b: Leukotriene B4 receptor 2b
ltc4s: Leukotriene C4 synthase
MAPK: Mitogen-activated protein kinases
MIA: Mirror-induced aggression
nos1: Nitric oxide synthase 1
NND: Nearest neighbour distances
NTD: Novel tank diving test
PCA: Principal component analysis
ptgr1: Prostaglandin reductase 1
rpS6: Ribosomal protein S6
scg5: Secretogranin V
SDMN: Social decision-making network
st6galnac3: ST6 (alpha-N-acetyl-neuraminyl-2,3-beta-galactosyl-1,3)-N-acetylgalactosaminide alpha-2,6-sialyltransferase 3
tlr4ba: Toll-like receptor 4b, duplicate a
tnfrsf1b: Tumour necrosis factor receptor superfamily member 1B
ublcp1: Ubiquitin-like domain-containing CTD phosphatase 1
wdr1: WD repeat domain 1
WGCNA: Weighted gene co-expression network analysis
xcr1a.1: Chemokine (C motif) receptor 1a, duplicate 1
xcr1b.1: Chemokine (C motif) receptor 1b, duplicate 1
