# Supplementary material for: Transcriptomic underpinnings of high and low mirror aggression zebrafish behaviours
Source: BMC Biol. 2022 May 2;20:97. doi: 10.1186/s12915-022-01298-z (PMC9059464; doi:10.1186/s12915-022-01298-z)
Supplement: Supplementary file 1 — Additional file 1: Fig. S1. High aggression zebrafish (HAZ) and low aggression zebrafish (LAZ) show similar locomotor activity, social behaviour and boldness. Fig. S2. Neurotranscriptomic differences of founder (F0) high aggression zebrafish (HAZ) and low aggression zebrafish (LAZ). Fig. S3. Behaviour of cohort 1 high aggression zebrafish (HAZ) and low aggression zebrafish (LAZ) during prolonged mirror exposure. Fig. S4. Sex-specific neurotranscriptomic differences between mirror-exposed high aggression zebrafish (HAZ) and low aggression zebrafish (LAZ). Fig. S5. Behaviour of the most aggressive high aggression zebrafish (HAZ) and least aggressive low aggression zebrafish (LAZ) during prolonged mirror exposure (cohort 2). Fig. S6. Ten most significant differentially expressed genes (DEGs) between female high aggression zebrafish (HAZf) and low aggression zebrafish (LAZf). Fig. S7. Ten most significant differentially expressed genes between male high aggression zebrafish (HAZm) and low aggression zebrafish (LAZm). Fig. S8. qPCR validation of RNAseq findings. Fig. S9. Morphological differences between male and female zebrafish independently of mirror aggression phenotype. Table 4. Differentially expressed genes between HAZf and LAZf associated with immune system related pathway terms obtained by DAVID pathway analysis. Table 5. Differentially expressed genes between HAZm and LAZm associated with immune system related pathway terms obtained by DAVID pathway analysis. Table 6. Differentially expressed genes involved in oxidation reduction processes for HAZf vs. LAZf and HAZm vs. LAZm obtained by DAVID pathway analysis. Table 7. Differentially expressed genes involved in membrane-related processes for HAZf vs. LAZf and HAZm vs. LAZm obtained by DAVID pathway analysis. Table 11. Primers used for real-time RT PCR to validate RNAseq findings. [file 12915_2022_1298_MOESM1_ESM.docx]

**Supplementary information**

Transcriptomic underpinnings of high- and low mirror aggression zebrafish behaviours

Florian Reichmann^1*^, Johannes Pilic^1^, Slave Trajanoski^3^, William HJ Norton^2,4*^

^1^ Division of Pharmacology, Otto Loewi Research Center, Medical University of Graz, Graz, Austria

^2^ Department of Genetics and Genome Biology, College of Life Sciences, University of Leicester, Leicester, UK

^3^Center for Medical Research, Medical University of Graz, Graz, Austria

^4^ELTE Eötvös Loránd University, Institute of Biology, Department of Genetics, Budapest, Hungary

*Corresponding authors:

Florian Reichmann, email: florian.reichmann@medunigraz.at

William HJ Norton, email: [whjn1@leicester.ac.uk](mailto:whjn1@leicester.ac.uk)

**Supplementary Figure 1:**

***Supplementary Figure 1. High aggression zebrafish (HAZ) and low aggression zebrafish (LAZ) show similar locomotor activity, social behaviour and boldness.*** *(a) Distance travelled, (b) velocity, (c) time spent immobile and (d) angular velocity of LAZ and HAZ during the open field test. n = 12/group. (e) Interindividual and (f) nearest neighbour distances of LAZ and HAZ during shoaling assays. n = 9 shoals/group. (g) Time spent in the social interaction zone of the visually-mediated social interaction test. n = 12/group. (h) Time spent in the near of a predator fish-shaped object in the boldness assay. n = 11-12/group.* *Source data and individual data values are available in Additional file 2.*

**Supplementary Figure 2:**

**
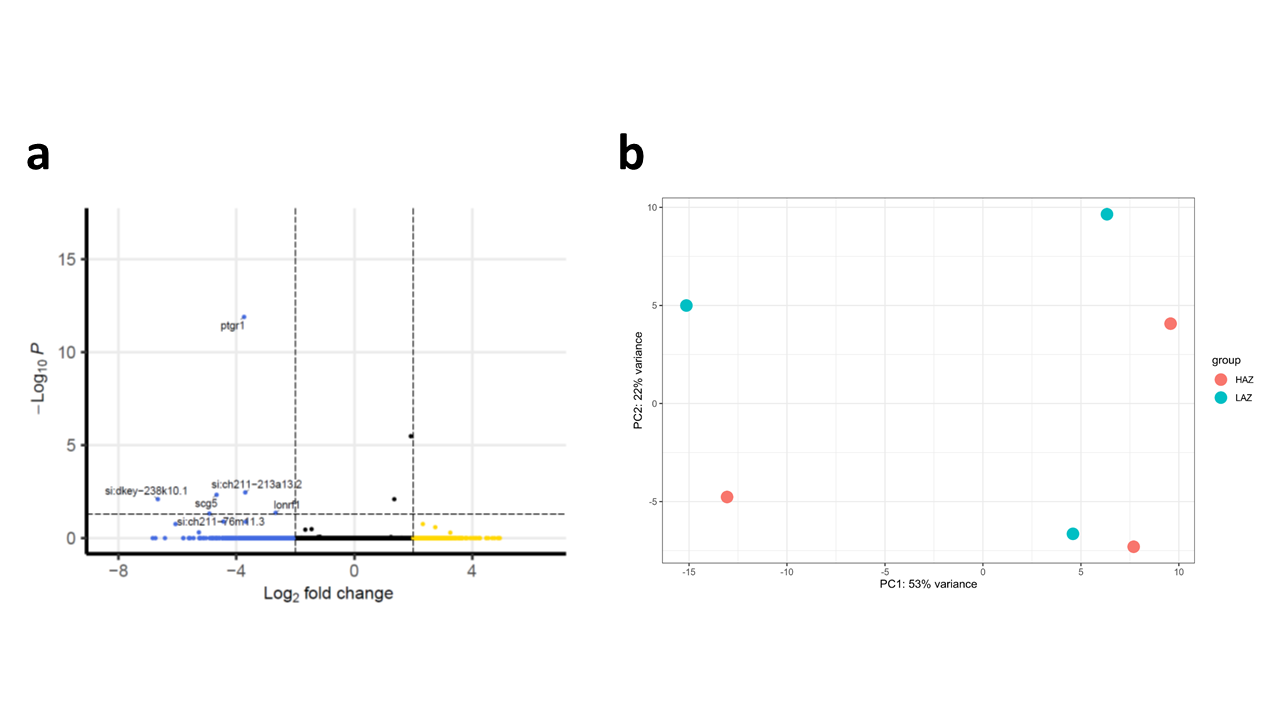
**

***Supplementary Figure 2. Neurotranscriptomic differences of founder (F0) high aggression zebrafish (HAZ) and low aggression zebrafish (LAZ).*** *(a) Volcano plot displaying differentially expressed genes (DEGs; padj < 0.05 and LFC > |2|) between HAZ and LAZ. n = 3/group. DEGs are labelled. (b) Principal component analysis plot of the top 200 most variable genes after differential expression analysis. n = 3/group. Golden dots in Volcano plots indicate genes upregulated in HAZ more than log fold change 2, blue dots represent genes downregulated in HAZ more than LFC -2 and black dots represent genes between these thresholds.* *Source data and individual data values are available at the ebrains data repository, DOI: 10.25493/VTP5-8J9 and in Additional file 2.*

**Supplementary Figure 3:**

**

**

***Supplementary Figure 3. Behaviour of cohort 1 high aggression zebrafish (HAZ) and low aggression zebrafish (LAZ) during prolonged mirror exposure.*** *(a) Time spent interacting with the mirror, (b) distance travelled and (c) time spent immobile of HAZ and LAZ exposed to a 1h mirror-induced aggression assay. Mann-Whitney-U test. n = 6/group. Data are presented as mean ± SEM. Source data and individual data values are available in Additional file 2.*

**Supplementary Figure 4:**

**
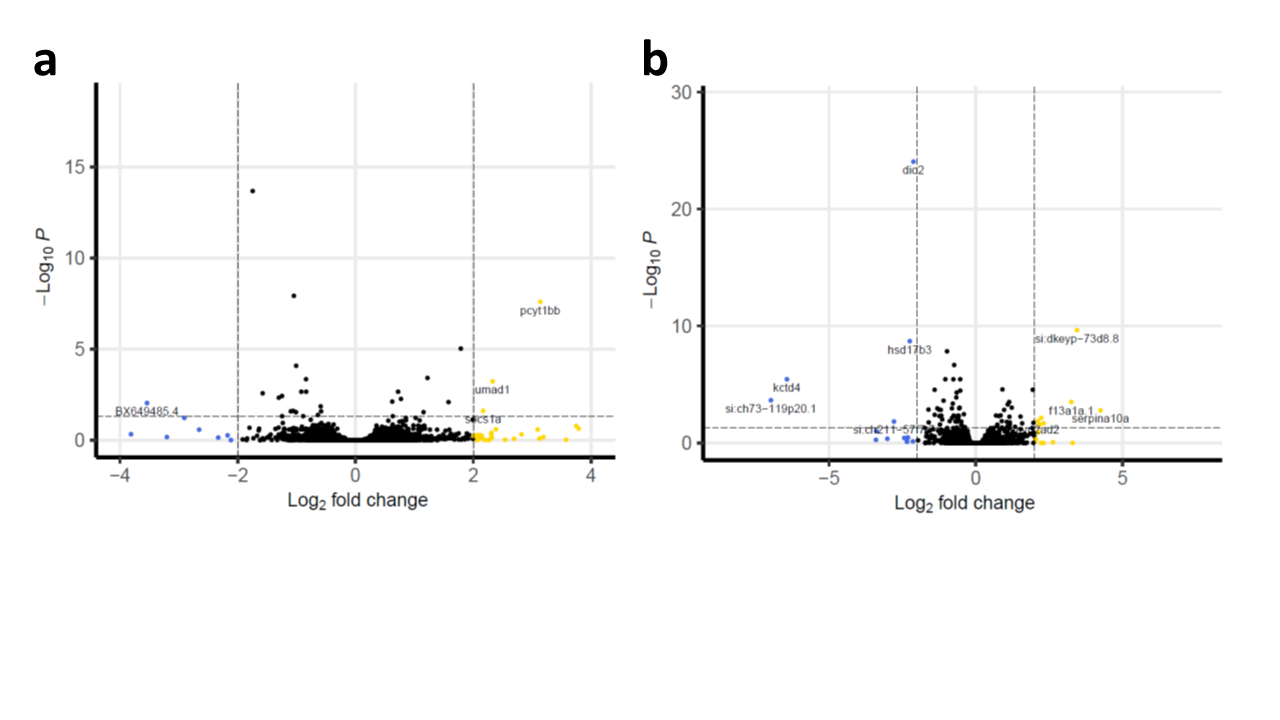
**

***Supplementary Figure 4. Sex-specific neurotranscriptomic differences between mirror-exposed high aggression zebrafish (HAZ) and low aggression zebrafish (LAZ).*** *(a) Volcano plot displaying differentially expressed genes (DEGs; padj < 0.05 and LFC > |2|) between male and female HAZ. DEGs are labelled. (b) Volcano plot displaying differentially expressed genes (DEGs; padj < 0.05 and LFC > |2|) between male and female LAZ. The most significant DEGs are labelled. Golden dots in Volcano plots indicate genes upregulated in females more than log fold change 2, blue dots represent genes downregulated in females more than LFC -2 and black dots represent genes between these thresholds. Source data and individual data values are available in Additional file 2.*

**Supplementary Figure 5:**

**

**

***Supplementary Figure 5. Behaviour of the most aggressive high aggression zebrafish (HAZ) and least aggressive low aggression zebrafish (LAZ) during prolonged mirror exposure (cohort 2).*** *(a) Time spent interacting with the mirror, (b) distance travelled and (c) time spent immobile of the 6 most aggressive male and female HAZ and the 6 least aggressive male and female LAZ out of 30 zebrafish/group exposed to a 1h mirror-induced aggression assay (cohort 2). Two-way ANOVA. n = 6/group. ***, main effect HAZ vs. LAZ, P < 0.001. Data are presented as mean ± SEM. Source data and individual data values are available in Additional file 2.*

**Supplementary Figure 6:**

***Supplementary Figure 6. Ten most significant differentially expressed genes (DEGs) between female high aggression zebrafish (HAZf) and low aggression zebrafish (LAZf).*** *(a) arsenite methyltransferase (as3mt), (b) solute carrier family 4 member 1 adaptor protein (slc4a1ap), (c) ADP-ribosylation factor-like 4ab (arl4ab), (d) prostaglandin reductase 1 (ptgr1), (e) saccharopine dehydrogenase a, tandem duplicate 1 (sccpdha.1), (f) WD repeat domain 1 (wdr1), (g) DiGeorge syndrome critical region gene 2 (dgcr2), (h) ubiquitin-like domain-containing CTD phosphatase 1 (ublcp1), (i) ST6 (alpha-N-acetyl-neuraminyl-2,3-beta-galactosyl-1,3)-N-acetylgalactosaminide alpha-2,6-sialyltransferase 3 (st6galnac3) and (j) acetoacetyl-CoA synthetase (aacs).* *DEGs were defined as padj < 0.05 and LFC > |2|. Data are presented as mean ± SEM. n = 6/group. Source data and individual data values are available in Additional file 2.*

**Supplementary Figure 7:**

***Supplementary Figure 7. Ten most significant differentially expressed genes between male high aggression zebrafish (HAZm) and low aggression zebrafish (LAZm).*** *(a) arsenite methyltransferase (as3mt), (b) prostaglandin reductase 1 (ptgr1), (c) ADP-ribosylation factor-like 4ab (arl4ab), (d) saccharopine dehydrogenase a, tandem duplicate 1 (sccpdha.1), (e) solute carrier family 4 member 1 adaptor protein (slc4a1ap), (f) ubiquitin-like domain-containing CTD phosphatase 1 (ublcp1), (g) DiGeorge syndrome critical region gene 2 (dgcr2), (h) F-box protein 31 (fbxo31), (i) acetoacetyl-CoA synthetase (aacs) and (j) ST6 (alpha-N-acetyl-neuraminyl-2,3-beta-galactosyl-1,3)-N-acetylgalactosaminide alpha-2,6-sialyltransferase 3 (st6galnac3). DEGs were defined as padj < 0.05 and LFC > |2|. Data are presented as mean ± SEM. Source data and individual data values are available in Additional file 2.*

**Supplementary Figure 8:**

**Supplementary Figure 8. qPCR validation of RNAseq findings.** *Six semi-randomly selected DEGs (as3mt, npy8br, cd164, ptgr1, fzd4, col12a1b) show similar log2 fold expression changes between HAZm and LAZm in RT-qPCR and RNAseq. Correlation analysis revealed a significant positive correlation (Pearson correlation coefficient of 0.869, p = 0.02). Source data and individual data values are available in Additional file 2.*

**Supplementary Figure 9:**

**

**

***Supplementary Figure 9. Morphological differences between male and female zebrafish independently of mirror aggression phenotype.*** *Width measurements of (a) 1V stripes, (b) 2V stripes and (c) X0 stripes and size measurements of (d) tail fins and (e) anal fins from stereomicroscopic images. Grayscale measurements of stereomicroscopic images from (f) whole body images, (g) 1D stripes, (h) 2V stripes and 1V stripes of male and female LAZ and HAZ. Two-way ANOVA. n = 6/group. ###, P<0.001; #, P<0.05 female vs. male main effect. Data are presented as mean ± SEM. Source data and individual data values are available in Additional file 2.*

**Table 4: Differentially expressed genes between HAZf and LAZf associated with immune system related pathway terms obtained by DAVID pathway analysis.**

| **ENSEMBL Gene ID** | **Gene Name** |
| --- | --- |
| ENSDARG00000017565 | IL2-inducible T-cell kinase(itk) |
| ENSDARG00000102525 | LCK proto-oncogene, Src family tyrosine kinase(lck) |
| ENSDARG00000032532 | cAMP-regulated phosphoprotein 19a(arpp19a) |
| ENSDARG00000055100 | chemokine (C-X-C motif) ligand 12b (stromal cell-derived factor 1)(cxcl12b) |
| ENSDARG00000044048 | prion protein b(prnpb) |
| ENSDARG00000068966 | si:ch211-261n11.7(si:ch211-261n11.7) |
| ENSDARG00000058753 | si:ch73-213k20.5(si:ch73-213k20.5) |
| ENSDARG00000031731 | si:ch73-27e22.6(si:ch73-27e22.6) |
| ENSDARG00000077115 | si:ch73-44m9.1(si:ch73-44m9.1) |
| ENSDARG00000058537 | si:dkey-102c8.2(si:dkey-102c8.2) |
| ENSDARG00000055831 | si:dkey-182g1.6(si:dkey-182g1.6) |
| ENSDARG00000071644 | si:dkey-19a16.7(si:dkey-19a16.7) |
| ENSDARG00000037362 | signal peptide, CUB domain, EGF-like 2(scube2) |
| ENSDARG00000070165 | tumor necrosis factor receptor superfamily, member 1B(tnfrsf1b) |
| ENSDARG00000098214 | uncharacterized LOC100329818(LOC100329818) |
| ENSDARG00000040640 | uncharacterized LOC101883645(LOC101883645) |
| ENSDARG00000091230 | uncharacterized LOC101886679(LOC101886679) |
| ENSDARG00000071643 | zgc:171490(zgc:171490) |
| ENSDARG00000100614 | zgc:171497(zgc:171497) |
| ENSDARG00000104592 | zgc:171601(zgc:171601) |
| ENSDARG00000058791 | zgc:171887(zgc:171887) |
| ENSDARG00000019601 | collagen, type XII, alpha 1b(col12a1b) |
| ENSDARG00000004105 | endothelium-specific receptor tyrosine kinase 1(tie1) |
| ENSDARG00000054542 | interleukin 12B, c(il12bc) |
| ENSDARG00000023203 | major histocompatibility complex class I LDA(mhc1lda) |
| ENSDARG00000076462 | neuron-derived neurotrophic factor , like(ndnfl) |
| ENSDARG00000017369 | sema domain, immunoglobulin domain (Ig), short basic domain, secreted, (semaphorin) 3D(sema3d) |
| ENSDARG00000097275 | si:ch211-147g22.4(si:ch211-147g22.4) |
| ENSDARG00000097726 | si:ch211-149e23.4(si:ch211-149e23.4) |
| ENSDARG00000078502 | si:ch211-150o23.3(si:ch211-150o23.3) |
| ENSDARG00000098216 | si:ch211-215e19.3(si:ch211-215e19.3) |
| ENSDARG00000092578 | si:ch211-222e20.4(si:ch211-222e20.4) |
| ENSDARG00000100887 | si:dkey-11f4.20(si:dkey-11f4.20) |
| ENSDARG00000099266 | vasorin a(vasna) |
| ENSDARG00000007787 | zgc:112965(zgc:112965) |
| ENSDARG00000021241 | zgc:165604(zgc:165604) |
| ENSDARG00000054847 | chemokine (C motif) receptor 1a, duplicate 1(xcr1a.1) |
| ENSDARG00000052988 | chemokine (C motif) receptor 1b, duplicate 1(xcr1b.1) |
| ENSDARG00000070755 | chemokine (C-C motif) receptor 11.1(ccr11.1) |
| ENSDARG00000095789 | chemokine (C-C motif) receptor 8.1(ccr8.1) |

**Table 5: Differentially expressed genes between HAZm and LAZm associated with immune system related pathway terms obtained by DAVID pathway analysis.**

| **ENSEMBL Gene ID** | **Gene Name** |
| --- | --- |
| ENSDARG00000090310 | SLAM family member 9-like(LOC100329398) |
| ENSDARG00000070378 | chemokine (C-C motif) ligand 35, duplicate 2(ccl35.2) |
| ENSDARG00000045453 | coagulation factor XIII, A1 polypeptide a, tandem duplicate 1(f13a1a.1) |
| ENSDARG00000019601 | collagen, type XII, alpha 1b(col12a1b) |
| ENSDARG00000054542 | interleukin 12B, c(il12bc) |
| ENSDARG00000054170 | leukotriene B4 receptor 2b(ltb4r2b) |
| ENSDARG00000023203 | major histocompatibility complex class I LDA(mhc1lda) |
| ENSDARG00000044048 | prion protein b(prnpb) |
| ENSDARG00000074510 | si:busm1-104n07.3(si:busm1-104n07.3) |
| ENSDARG00000097275 | si:ch211-147g22.4(si:ch211-147g22.4) |
| ENSDARG00000097726 | si:ch211-149e23.4(si:ch211-149e23.4) |
| ENSDARG00000068966 | si:ch211-261n11.7(si:ch211-261n11.7) |
| ENSDARG00000089901 | si:ch211-261n11.8(si:ch211-261n11.8) |
| ENSDARG00000068637 | si:ch211-281l24.3(si:ch211-281l24.3) |
| ENSDARG00000058753 | si:ch73-213k20.5(si:ch73-213k20.5) |
| ENSDARG00000031731 | si:ch73-27e22.6(si:ch73-27e22.6) |
| ENSDARG00000077115 | si:ch73-44m9.1(si:ch73-44m9.1) |
| ENSDARG00000058537 | si:dkey-102c8.2(si:dkey-102c8.2) |
| ENSDARG00000071644 | si:dkey-19a16.7(si:dkey-19a16.7) |
| ENSDARG00000068993 | zgc:153631(zgc:153631) |
| ENSDARG00000021241 | zgc:165604(zgc:165604) |
| ENSDARG00000071643 | zgc:171490(zgc:171490) |
| ENSDARG00000100614 | zgc:171497(zgc:171497) |
| ENSDARG00000058791 | zgc:171887(zgc:171887) |
| ENSDARG00000091650 | IGF-like family receptor 1(igflr1) |
| ENSDARG00000035677 | bone morphogenetic protein 8a(bmp8a) |
| ENSDARG00000026925 | nitric oxide synthase 2a, inducible(nos2a) |
| ENSDARG00000091280 | si:ch211-66k16.27(si:ch211-66k16.27) |
| ENSDARG00000091926 | si:dkey-103e21.5(si:dkey-103e21.5) |
| ENSDARG00000052988 | chemokine (C motif) receptor 1b, duplicate 1(xcr1b.1) |
| ENSDARG00000070755 | chemokine (C-C motif) receptor 11.1(ccr11.1) |
| ENSDARG00000095789 | chemokine (C-C motif) receptor 8.1(ccr8.1) |

**Table 6: Differentially expressed genes involved in oxidation reduction processes for HAZf vs. LAZf and HAZm vs. LAZm obtained by DAVID pathway analysis.**

| **ENSEMBL Gene ID** | **Gene Name** |
| --- | --- |
| ENSDARG00000042827 | 2-aminoethanethiol (cysteamine) dioxygenase a(adoa) |
| ENSDARG00000057273 | arachidonate 5-lipoxygenase a(alox5a) |
| ENSDARG00000002347 | cytochrome P450, family 11, subfamily A, polypeptide 1(cyp11a1) |
| ENSDARG00000098803 | cytochrome P450, family 2, subfamily AA, polypeptide 8(cyp2aa8) |
| ENSDARG00000102981 | cytochrome P450, family 2, subfamily K, polypeptide16(cyp2k16) |
| ENSDARG00000070021 | cytochrome P450, family 3, subfamily C, polypeptide 4(cyp3c4) |
| ENSDARG00000038834 | electron-transferring-flavoprotein dehydrogenase(etfdh) |
| ENSDARG00000015228 | endoplasmic reticulum oxidoreductase alpha(ero1a) |
| ENSDARG00000012535 | eosinophil peroxidase(epx) |
| ENSDARG00000019986 | glyoxylate reductase/hydroxypyruvate reductase b(grhprb) |
| ENSDARG00000061301 | guanosine monophosphate reductase 2(gmpr2) |
| ENSDARG00000012016 | hydroxyprostaglandin dehydrogenase 15-(NAD)(hpgd) |
| ENSDARG00000045553 | hydroxysteroid (17-beta) dehydrogenase 2(hsd17b2) |
| ENSDARG00000026925 | nitric oxide synthase 2a, inducible(nos2a) |
| ENSDARG00000031976 | nitric oxide synthase 2b, inducible(nos2b) |
| ENSDARG00000103692 | prolyl 4-hydroxylase, alpha polypeptide III(p4ha3) |
| ENSDARG00000024877 | prostaglandin reductase 1(ptgr1) |
| ENSDARG00000087017 | prostaglandin reductase 1-like(LOC566996) |
| ENSDARG00000070673 | prostaglandin reductase 2(ptgr2) |
| ENSDARG00000010276 | prostaglandin-endoperoxide synthase 2b(ptgs2b) |
| ENSDARG00000010555 | pyruvate dehydrogenase (lipoamide) alpha 1b(pdha1b) |
| ENSDARG00000078069 | ribonucleotide reductase M2 polypeptide(rrm2) |
| ENSDARG00000020711 | ribonucleotide reductase regulatory subunit M2(rrm2) |
| ENSDARG00000075766 | saccharopine dehydrogenase a(sccpdha) |
| ENSDARG00000061758 | SH3 and PX domains 2Ab(sh3pxd2ab) |
| ENSDARG00000032816 | transmembrane 7 superfamily member 2(tm7sf2) |
| ENSDARG00000019838 | UDP-glucose 6-dehydrogenase(ugdh) |
| ENSDARG00000054934 | zgc:101765(zgc:101765) |
| ENSDARG00000017038 | zgc:152670(zgc:152670) |
| ENSDARG00000061481 | zgc:163022(zgc:163022) |
| ENSDARG00000075053 | zgc:174379(zgc:174379) |

DEGs present in both comparisons are highlighted in green, those exclusively present in HAZf vs LAZf are highlighted in gold and those exclusively present in HAZm vs LAZm are highlighted in blue.

**Table 7: Differentially expressed genes involved in membrane-related processes for HAZf vs. LAZf and HAZm vs. LAZm** **obtained by DAVID pathway analysis.**

| ENSDARG00000101130 | aarF domain containing kinase 4(adck4) |
| --- | --- |
| ENSDARG00000041173 | adrenoceptor alpha 1Bb(adra1bb) |
| ENSDARG00000100159 | angiopoietin-like 1b(angptl1b) |
| ENSDARG00000003808 | aquaporin 3a(aqp3a) |
| ENSDARG00000022832 | BCL2/adenovirus E1B interacting protein 4(bnip4) |
| ENSDARG00000035677 | bone morphogenetic protein 8a(bmp8a) |
| ENSDARG00000060637 | calsyntenin 2(clstn2) |
| ENSDARG00000013628 | CD164 molecule, sialomucin(cd164) |
| ENSDARG00000087245 | CD209 antigen-like protein D(LOC101884526) |
| ENSDARG00000054847 | chemokine (C motif) receptor 1a, duplicate 1(xcr1a.1) |
| ENSDARG00000052988 | chemokine (C motif) receptor 1b, duplicate 1(xcr1b.1) |
| ENSDARG00000070755 | chemokine (C-C motif) receptor 11.1(ccr11.1) |
| ENSDARG00000095789 | chemokine (C-C motif) receptor 8.1(ccr8.1) |
| ENSDARG00000055559 | cholinergic receptor, nicotinic, alpha 6(chrna6) |
| ENSDARG00000030723 | claudin 11b(cldn11b) |
| ENSDARG00000015955 | claudin c(cldnc) |
| ENSDARG00000006580 | claudin d(cldnd) |
| ENSDARG00000069503 | claudin h(cldnh) |
| ENSDARG00000003925 | connexin 28.6(cx28.6) |
| ENSDARG00000076789 | connexin 32.2(cx32.2) |
| ENSDARG00000103704 | corticotropin releasing hormone receptor 2(crhr2) |
| ENSDARG00000087013 | cubilin (intrinsic factor-cobalamin receptor)(cubn) |
| ENSDARG00000100436 | cyclic nucleotide-gated cation channel-like(LOC103911065) |
| ENSDARG00000098803 | cytochrome P450, family 2, subfamily AA, polypeptide 8(cyp2aa8) |
| ENSDARG00000102981 | cytochrome P450, family 2, subfamily K, polypeptide16(cyp2k16) |
| ENSDARG00000070021 | cytochrome P450, family 3, subfamily C, polypeptide 4(cyp3c4) |
| ENSDARG00000088693 | DC-STAMP domain containing 2(dcst2) |
| ENSDARG00000042962 | DiGeorge syndrome critical region gene 2(dgcr2) |
| ENSDARG00000038834 | electron-transferring-flavoprotein dehydrogenase(etfdh) |
| ENSDARG00000015228 | endoplasmic reticulum oxidoreductase alpha(ero1a) |
| ENSDARG00000087457 | endothelial cell surface expressed chemotaxis and apoptosis regulator(ecscr) |
| ENSDARG00000004105 | endothelium-specific receptor tyrosine kinase 1(tie1) |
| ENSDARG00000012535 | eosinophil peroxidase(epx) |
| ENSDARG00000102701 | eva-1 homolog Ba (C. elegans)(eva1ba) |
| ENSDARG00000063191 | exostosin-like glycosyltransferase 2(extl2) |
| ENSDARG00000105362 | Fas apoptotic inhibitory molecule 2b(faim2b) |
| ENSDARG00000104117 | frizzled class receptor 4(fzd4) |
| ENSDARG00000038569 | frizzled class receptor 8b(fzd8b) |
| ENSDARG00000062934 | G protein-coupled receptor 12(gpr12) |
| ENSDARG00000079826 | G protein-coupled receptor 174(gpr174) |
| ENSDARG00000102758 | gamma-glutamyl hydrolase(LOC553228) |
| ENSDARG00000025275 | globoside alpha-1,3-N-acetylgalactosaminyltransferase 1, like 1(gbgt1l1) |
| ENSDARG00000062688 | glycoprotein (transmembrane) nmb(gpnmb) |
| ENSDARG00000018329 | guanylyl cyclase 2(gc2) |
| ENSDARG00000091650 | IGF-like family receptor 1(igflr1) |
| ENSDARG00000078415 | leucine rich repeat containing 3(lrrc3) |
| ENSDARG00000054170 | leukotriene B4 receptor 2b(ltb4r2b) |
| ENSDARG00000062598 | leukotriene C4 synthase(ltc4s) |
| ENSDARG00000074638 | lipoxygenase homology domains 1b(loxhd1b) |
| ENSDARG00000043093 | macrophage expressed 1, tandem duplicate 2(mpeg1.2) |
| ENSDARG00000101379 | major facilitator superfamily domain containing 1(mfsd1) |
| ENSDARG00000035909 | major facilitator superfamily domain containing 2ab(mfsd2ab) |
| ENSDARG00000023203 | major histocompatibility complex class I LDA(mhc1lda) |
| ENSDARG00000055852 | melatonin receptor 1A b(mtnr1ab) |
| ENSDARG00000012057 | melatonin receptor type 1A like(mtnr1al) |
| ENSDARG00000043802 | membrane-spanning 4-domains, subfamily A, member 17A.9(ms4a17a.9) |
| ENSDARG00000088546 | myc target 1b(myct1b) |
| ENSDARG00000005780 | neuropeptide Y receptor Y8b(npy8br) |
| ENSDARG00000100604 | olfactory receptor C family, w1(olfcw1) |
| ENSDARG00000044861 | opsin 1 (cone pigments), long-wave-sensitive, 2(opn1lw2) |
| ENSDARG00000052056 | parietopsin(parietopsin) |
| ENSDARG00000006109 | Pim proto-oncogene, serine/threonine kinase, related 185(pimr185) |
| ENSDARG00000023587 | potassium channel, subfamily K, member 5a(kcnk5a) |
| ENSDARG00000044048 | prion protein b(prnpb) |
| ENSDARG00000103692 | prolyl 4-hydroxylase, alpha polypeptide III(p4ha3) |
| ENSDARG00000035161 | protein kinase domain containing, cytoplasmic a(pkdcca) |
| ENSDARG00000089641 | protein phosphatase, Mg2+/Mn2+ dependent, 1Lb(ppm1lb) |
| ENSDARG00000101232 | protocadherin 1 gamma 2(pcdh1g2) |
| ENSDARG00000088475 | protocadherin 1 gamma b 9(pcdh1gb9) |
| ENSDARG00000004952 | radical S-adenosyl methionine domain containing 2(rsad2) |
| ENSDARG00000100265 | Rh family, C glycoprotein b(rhcgb) |
| ENSDARG00000078069 | ribonucleotide reductase M2 polypeptide(rrm2) |
| ENSDARG00000020711 | ribonucleotide reductase regulatory subunit M2(rrm2) |
| ENSDARG00000024827 | ring finger protein 150(rnf150) |
| ENSDARG00000009123 | selectin E(sele) |
| ENSDARG00000006307 | shisa family member 4(shisa4) |
| ENSDARG00000074510 | si:busm1-104n07.3(si:busm1-104n07.3) |
| ENSDARG00000097890 | si:ch1073-100f3.2(si:ch1073-100f3.2) |
| ENSDARG00000077219 | si:ch211-106h4.6(si:ch211-106h4.6) |
| ENSDARG00000093521 | si:ch211-126g16.11(si:ch211-126g16.11) |
| ENSDARG00000077090 | si:ch211-127b11.1(si:ch211-127b11.1) |
| ENSDARG00000097275 | si:ch211-147g22.4(si:ch211-147g22.4) |
| ENSDARG00000097726 | si:ch211-149e23.4(si:ch211-149e23.4) |
| ENSDARG00000078502 | si:ch211-150o23.3(si:ch211-150o23.3) |
| ENSDARG00000093044 | si:ch211-161h7.5(si:ch211-161h7.5) |
| ENSDARG00000063519 | si:ch211-176g13.7(si:ch211-176g13.7) |
| ENSDARG00000077572 | si:ch211-193k19.2(si:ch211-193k19.2) |
| ENSDARG00000012496 | si:ch211-213o11.11(si:ch211-213o11.11) |
| ENSDARG00000020952 | si:ch211-214j8.1(si:ch211-214j8.1) |
| ENSDARG00000097453 | si:ch211-225b7.5(si:ch211-225b7.5) |
| ENSDARG00000097693 | si:ch211-248a14.8(si:ch211-248a14.8) |
| ENSDARG00000068966 | si:ch211-261n11.7(si:ch211-261n11.7) |
| ENSDARG00000089901 | si:ch211-261n11.8(si:ch211-261n11.8) |
| ENSDARG00000046091 | si:ch211-283g2.1(si:ch211-283g2.1) |
| ENSDARG00000069978 | si:ch211-67e16.2(si:ch211-67e16.2) |
| ENSDARG00000099257 | si:ch73-119p20.1(si:ch73-119p20.1) |
| ENSDARG00000069323 | si:ch73-122g19.1(si:ch73-122g19.1) |
| ENSDARG00000056045 | si:ch73-139e5.4(si:ch73-139e5.4) |
| ENSDARG00000017722 | si:ch73-151m17.5(si:ch73-151m17.5) |
| ENSDARG00000058753 | si:ch73-213k20.5(si:ch73-213k20.5) |
| ENSDARG00000031731 | si:ch73-27e22.6(si:ch73-27e22.6) |
| ENSDARG00000077115 | si:ch73-44m9.1(si:ch73-44m9.1) |
| ENSDARG00000100887 | si:dkey-11f4.20(si:dkey-11f4.20) |
| ENSDARG00000086236 | si:dkey-163m14.7(si:dkey-163m14.7) |
| ENSDARG00000055831 | si:dkey-182g1.6(si:dkey-182g1.6) |
| ENSDARG00000094265 | si:dkey-183n20.15(si:dkey-183n20.15) |
| ENSDARG00000071644 | si:dkey-19a16.7(si:dkey-19a16.7) |
| ENSDARG00000101201 | si:dkey-207b20.2(si:dkey-207b20.2) |
| ENSDARG00000001906 | si:dkey-247m21.3(si:dkey-247m21.3) |
| ENSDARG00000069540 | si:dkey-30c15.2(si:dkey-30c15.2) |
| ENSDARG00000099117 | si:dkey-33i11.4(si:dkey-33i11.4) |
| ENSDARG00000093997 | si:dkey-9i23.15(si:dkey-9i23.15) |
| ENSDARG00000095407 | si:dkey-9i23.8(si:dkey-9i23.8) |
| ENSDARG00000038112 | si:dkeyp-59c12.1(si:dkeyp-59c12.1) |
| ENSDARG00000094336 | si:dkeyp-82a1.6(si:dkeyp-82a1.6) |
| ENSDARG00000010047 | sialidase 3 (membrane sialidase), tandem duplicate 2(neu3.2) |
| ENSDARG00000017960 | sideroflexin 2(sfxn2) |
| ENSDARG00000090310 | SLAM family member 9-like(LOC100329398) |
| ENSDARG00000061414 | SLX4 structure-specific endonuclease subunit homolog (S. cerevisiae)(slx4) |
| ENSDARG00000091252 | solute carrier family 22 (organic anion transporter), member 7b, |
|  | tandem duplicate 2(slc22a7b.2) |
| ENSDARG00000074860 | solute carrier family 5 (sodium/choline cotransporter), member 7a(slc5a7a) |
| ENSDARG00000100919 | solute carrier family 5 (sodium/glucose cotransporter), member 2(slc5a2) |
| ENSDARG00000067713 | sorting nexin 18a(snx18a) |
| ENSDARG00000058473 | ST6 (alpha-N-acetyl-neuraminyl-2,3-beta-galactosyl-1,3)-N-acetylgalactosaminide |
|  | alpha-2,6-sialyltransferase 3(st6galnac3) |
| ENSDARG00000039455 | tetraspanin 15(tspan15) |
| ENSDARG00000044767 | tetraspanin-10(LOC799194) |
| ENSDARG00000079122 | thioredoxin-related transmembrane protein 2a(tmx2a) |
| ENSDARG00000019742 | toll-like receptor 4b, duplicate a(tlr4ba) |
| ENSDARG00000090119 | toll-like receptor 8a(tlr8a) |
| ENSDARG00000055185 | transmembrane 4 L six family member 5(tm4sf5) |
| ENSDARG00000032816 | transmembrane 7 superfamily member 2(tm7sf2) |
| ENSDARG00000045525 | transmembrane and coiled-coil domain family 3(tmcc3) |
| ENSDARG00000056628 | transmembrane protein 170A(tmem170a) |
| ENSDARG00000091058 | transmembrane protein 173(tmem173) |
| ENSDARG00000059247 | transmembrane protein 54a(tmem54a) |
| ENSDARG00000061723 | transmembrane protein 64(tmem64) |
| ENSDARG00000074506 | transmembrane protein 94(tmem94) |
| ENSDARG00000070165 | tumor necrosis factor receptor superfamily, member 1B(tnfrsf1b) |
| ENSDARG00000054835 | UDP glucuronosyltransferase 5 family, polypeptide F1(ugt5f1) |
| ENSDARG00000098214 | uncharacterized LOC100329818(LOC100329818) |
| ENSDARG00000040640 | uncharacterized LOC101883645(LOC101883645) |
| ENSDARG00000091230 | uncharacterized LOC101886679(LOC101886679) |
| ENSDARG00000093546 | uncharacterized LOC798290(LOC798290) |
| ENSDARG00000099266 | vasorin a(vasna) |
| ENSDARG00000086183 | vitamin K epoxide reductase complex, subunit 1(vkorc1) |
| ENSDARG00000089361 | wu:fb59d01(wu:fb59d01) |
| ENSDARG00000020443 | XK, Kell blood group complex subunit-related family, member 6a(xkr6a) |
| ENSDARG00000007787 | zgc:112965(zgc:112965) |
| ENSDARG00000068993 | zgc:153631(zgc:153631) |
| ENSDARG00000093006 | zgc:154040(zgc:154040) |
| ENSDARG00000074663 | zgc:162183(zgc:162183) |
| ENSDARG00000061481 | zgc:163022(zgc:163022) |
| ENSDARG00000021241 | zgc:165604(zgc:165604) |
| ENSDARG00000078551 | zgc:171242(zgc:171242) |
| ENSDARG00000071643 | zgc:171490(zgc:171490) |
| ENSDARG00000100614 | zgc:171497(zgc:171497) |
| ENSDARG00000104592 | zgc:171601(zgc:171601) |
| ENSDARG00000058791 | zgc:171887(zgc:171887) |
| ENSDARG00000077638 | zgc:171965(zgc:171965) |
| ENSDARG00000088524 | zgc:77651(zgc:77651) |
| ENSDARG00000043482 | zgc:86586(zgc:86586) |
| ENSDARG00000097576 | zgc:92912(zgc:92912) |
| ENSDARG00000042872 | zinc finger, DHHC-type containing 8a(zdhhc8a) |

*DEGs present in both comparisons are highlighted in green, those exclusively present in HAZf vs LAZf are highlighted in gold and those exclusively present in HAZm vs LAZm are highlighted in blue.*

**Table 11: Primers used for real-time RT PCR to validate RNAseq findings.**

| Gene symbol | Sequence forward primer (5’→3’) | Sequence reverse primer (5’→3’) |
| --- | --- | --- |
| as3mt | GTGTTCTGAGGGAGGCGTAT | TGAGGCACTCGCCCCATAAA |
| npy8br | TTGCCTACCATTGGCGCTTA | CGCTTTGAGCCTTTGGCTTT |
| cd164 | TGCTACCACCAATGCTACCAC | GCGTCAAATGTGGACGTCTT |
| ptgr1 | ACTGCTACTTTGAGAGCGTGGG | ACACAGCAATACGCCCCAAA |
| fzd4 | ATCTGGTGGGAAACGTGCTA | AGACGGAGCATAAGAAGAACTTCAG |
| col12a1b | AAAGCATGGTAGGACTGGAGC | AGACATTCTCTGGCCTTCTCC |
| actb1 | TTCAGTGCACGCTGAGAAGA | CCAACCATCACTCCCTGATGT |
| rpl13a | TCTGGAGGACTGTAAGAGGTATGC | AGACGCACAATCTTGAGAGCAG |

*Abbreviations: as3mt…arsenite methyltransferase, npy8br…neuropeptide Y receptor Y8b, cd164…CD164 molecule, sialomucin, ptgr1…prostaglandin reductase 1, fzd4…frizzled class receptor 4, col12a1b…collagen, type XII, actb1…actin beta1, rpl13a…ribosomal protein L13a*
